# Telomere length dynamics in adults living with HIV: A systematic review

**DOI:** 10.1186/s12879-026-13243-4

**Published:** 2026-04-18

**Authors:** Myrix Karen Yabo Massamba, Lorna Madurai, Derseree Archary, Sharana Mahomed

**Affiliations:** 1 Department of Medical Microbiology, School of Laboratory Medicine & Medical Science, University of KwaZulu-Natal, Durban, South Africa; 2 Universal Pathology Laboratory, Durban, South Africa; 3 Centre for the AIDS Programme of Research in South Africa (CAPRISA), KwaZulu-Natal, Durban, South Africa

**Keywords:** HIV, Telomere length, Antiretroviral therapy, Accelerated ageing, Age-related diseases

## Abstract

**Background:**

HIV infection is associated with accelerated biological ageing, with telomere shortening serving as a key biomarker. Although numerous studies have investigated telomere dynamics in people living with HIV (PLWH), results vary depending on study design, population characteristics, treatment exposure, and measurement method. Herein, we summarize existing literature on telomere length (TL) dynamics in PLWH.

**Methods:**

A comprehensive search for original articles was conducted (07/03/2026) across four databases: PubMed, Scopus, Google Scholar, and ScienceDirect. Studies measuring TL quantitatively in PLWH using validated methods were included. Relevant literature was screened and data on study design, population characteristics, key findings and method of telomere measurement were extracted. A narrative synthesis approach was used to describe trends and study quality was assessed using the GRADE framework. PROSPERO ID: CRD420251241365.

**Results:**

Fifty-nine studies met the inclusion criteria. Most (37/59) were cross-sectional, with fewer (13/59) longitudinal studies assessing within-person telomere dynamics. Across studies, HIV infection was associated with shorter TL, although the magnitude of association varied by sex, antiretroviral therapy status, cell type, co-infections, and measurement technique. Telomere attrition appeared more pronounced around the time of seroconversion and in untreated or rapidly progressing individuals. Initiation of antiretroviral therapy (ART) has been associated with partial recovery of TL, although outcomes appear to vary according to regimen type and timing of initiation. Adjustment for potential confounders varied considerably across studies.

**Conclusions:**

The available evidence indicates a general association between HIV infection and shorter TL across diverse populations and methodological approaches. However, the predominance of observational and cross-sectional designs limits causal inference and precise characterization of telomere trajectories. While shorter telomeres have been linked to age-related comorbidities in PLWH, their clinical utility as a biomarker remains uncertain. Well-designed longitudinal studies with repeated measurements would allow more precise characterization of individual telomere trajectories across the course of HIV infection and treatment.

**Clinical trial number:**

Not applicable.

**Supplementary Information:**

The online version contains supplementary material available at 10.1186/s12879-026-13243-4.

## Introduction

Once synonymous with death, HIV is now a chronic, treatable condition, a transformation driven by the introduction of antiretroviral therapy (ART). However, researchers have noted a shift in mortality and morbidity patterns among people living with HIV (PLWH), with a growing emphasis on non-AIDS-related conditions [1]. The process of ageing is largely characterized by chronic inflammation and modifications to the innate and adaptive immune responses [2, 3]. This includes impaired thymic output due to age-related thymus shrinkage [4], increased levels of immune activation markers [5], a skew toward more exhausted memory T cell subsets with a history of repeated proliferation [3], and shortened telomeres [6]. When observed together, these changes mark the immune system as being in a state of senescence, thereby increasing vulnerability to age-related diseases. With an immune-compromising virus like HIV, this process is further accelerated by persistent low-level immune activation and chronic inflammation resulting from incomplete suppression of viral replication by ART [7–10]. Consequently, PLWH remain at a higher risk of developing cancers, metabolic, bone, cardiovascular, neurodegenerative, and other non-AIDS-related diseases much earlier than the general population [11–14].

Considering that chronological age is simply the passage of time, it is limited in capturing the function and health status of one’s immune system [15]. Therefore, in an effort to explore HIV driven immune ageing, various biomarkers have been considered, with the aim of improving management and potentially the overall quality of life of PLWH. Due to its inverse relationship with age, telomere length (TL) has long been recognized as both a biomarker of ageing and an indicator of mortality risk [6, 16]. Telomeres are short, repetitive DNA sequences, bound by specialized protein complexes, that cap the ends of linear chromosomes [17]. They maintain genomic stability by protecting chromosome ends from degradation and inappropriate end-to-end fusion [18, 19]. Given that DNA polymerase is unable to replicate DNA *de novo* [20], telomeres are gradually eroded with each round of cell division [21]. When telomeres become critically short, their protective capacity is compromised, leading to the activation of DNA damage response pathways. Eventually the cell enters a vegetative state known as senescence, where it is no longer dividing but still metabolically active [22]. This replicative limit, known as the Hayflick limit, reflects the natural endpoint of a cell’s proliferative capacity [23]. Senescent cells release various signaling molecules [24] and their accumulation is a core driver of age-related immune system decline [25]. Beyond the natural effects of ageing, TL is influenced by several intrinsic and extrinsic factors, such as sex, stress, lifestyle, and chronic illness [25]. Many studies have further linked shortened telomeres to a wide range of age-related disorders [26–30].

A growing number of research has been conducted to explore HIV-induced accelerated ageing through analyzing TL. In a recent systematic review exploring TL across various infectious diseases, HIV stood out as the most frequently studied pathogen, highlighting its relevance in immune ageing research [31]. Despite growing interest, studies on TL in PLWH are heterogeneous in design, population characteristics, measurement methods, and immune cell subsets analyzed. This variability has made it challenging to draw overarching conclusions regarding the extent and drivers of telomere shortening in this population. A comprehensive review of existing evidence is therefore needed to clarify current understanding, identify gaps, and inform future research and clinical practice; therefore, this systematic review aims to summarize current knowledge on TL dynamics in adults living with HIV.

## Methodology

The review protocol was developed in accordance with the Preferred Reporting Items for Systematic Reviews and Meta-analyses Protocols (PRISMA-P) guidelines [32]. Ethical approval was not required, as the review used publicly available secondary data. This systematic review was registered with PROSPERO (ID: CRD420251241365).

### Search strategy

On March 7th, 2026, an in-depth search was conducted utilizing four electronic databases: PubMed, Google Scholar, Scopus, and ScienceDirect. The terms “telomere length” and “HIV” were used in combination with Boolean operators and database-specific filters to maximize the retrieval of relevant studies. Additional synonyms and related terms (e.g., “telomere shortening,” “telomere attrition,” “telomeres,” “PLWH,” and “human immunodeficiency virus”) were incorporated into the search strings, as detailed in Supplementary Table 1. The reference lists of all included papers and relevant reviews were screened to identify any other eligible literature. All records were imported into EndNote (version 2025) for citation management, duplicate removal, and screening.

### Study selection

Study selection was facilitated by two independent reviewers, where the title, abstract and full texts of each record were screened according to the predefined inclusion and exclusion criteria. Any discrepancies encountered during the selection process were resolved by a third reviewer.

### Inclusion criteria

- **Study design**: Cross-sectional, longitudinal, case-control, cohort studies, and clinical trials.
- **Population**: Human adults living with HIV (including young adults with perinatally acquired HIV aged ≥18).
- **Comparators**: Depending on study design, comparators may include HIV-negative individuals, age/sex matched individuals with normal TL, HIV-positive individuals without the outcome under investigation (e.g. frailty) or stratified by ART status (e.g. ART naïve vs. ART experienced) and within-person pre-infection baselines in longitudinal studies.
- **Outcomes**: Studies that measure and report TL quantitatively or evaluate TL dynamics over time in PLWH, using validated methods such as qPCR (T/S ratio, monochrome multiplex), TRF (Terminal Restriction Fragment) analysis, FISH (Flow-FISH, Q-FISH), STELA (Single Telomere Length Analysis), TeSLA (Telomere Shortest Length Assay) and DNAmTL (DNA methylation based estimator of telomere length).
- Original research articles.
- Research published between 1996 – August 2025.
- Articles published in English.

### Exclusion criteria

- **Study design**: Reviews, meta-analyses, editorials, commentaries, protocols, conference abstracts, and book chapters.
- **Population**: Non-human studies (e.g., animal, in vitro, ex vivo without human participants) and studies including children <18 years old.
- **Confounders**: Studies where major comorbidities confound HIV-telomere dynamics (e.g. active Hepatitis C virus coinfection, active Tuberculosis or uncontrolled cancer).
- **Outcome**: Studies that do not quantitatively measure TL or assess telomere dynamics in PLWH, including those that only report telomerase activity, telomere-related gene expression, or other indirect markers, or that use non-validated methods or provide insufficient data to extract TL outcomes.
- Articles not published in English.

### Data extraction

Data extraction was performed by the first author using a standardized extraction form and independently verified by a second reviewer to ensure accuracy and consistency. Extracted variables included:

- Study characteristics (author, year, country, study design, sample size).
- Participant characteristics (age, sex, ART status, biological factors).
- Method of TL measurement.
- Comparator groups.
- Main study findings and key outcomes.
- Statistical adjustments for confounders.

### Data synthesis

Given the anticipated heterogeneity across studies, a narrative synthesis approach was employed. Potential sources of heterogeneity included variability in TL measurement modalities (e.g., qPCR, TRF, DNAmTL), differences in specimen cell type, study design, population characteristics (e.g., ART status and duration of infection), as well as variation in analytical approaches, particularly the adjustment for key confounders. Under these conditions, pooled effect estimates, heterogeneity statistics, forest plots, and publication-bias assessments would risk producing misleading summary estimates. Extracted data were therefore stratified across key study characteristics, with findings synthesized through structured summaries to enable systematic comparison and identify patterns, trends, and gaps in the literature.

### Quality assessment

The Grading of Recommendations Assessment, Development, and Evaluation (GRADE) approach was used to assess the quality and strength of a body of evidence as high, moderate, low, or very low. Risk of bias, study limitations, consistency of findings, and precision of estimates were evaluated systematically [33].

## Results

**Fig. 1:**
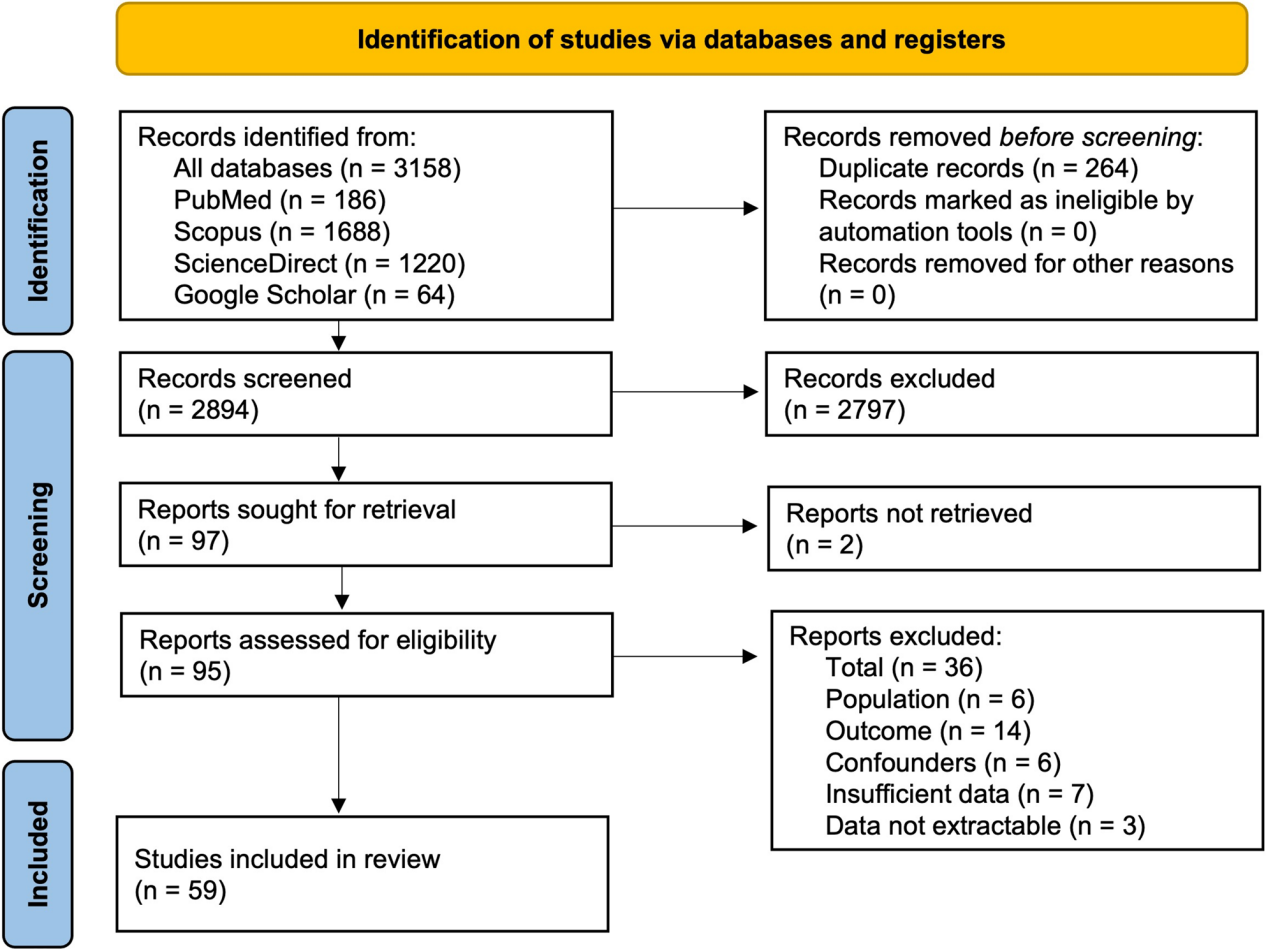
PRISMA flow diagram showing selection and screening of relevant studies evaluating telomere length dynamics in PLWH

The initial search from all four databases yielded 3158 records. After removing duplicates and irrelevant work, 97 papers were identified for full-text retrieval. Where access to full texts was restricted, digital copies were obtained through institutional access provided by the University of KwaZulu-Natal library. In total, 95 articles were successfully retrieved and assessed using the predefined inclusion and exclusion criteria, as illustrated in the PRISMA flow diagram **(**Fig. 1**)**. All research included in this review is summarized in Table 1.

The 59 included studies demonstrated substantial heterogeneity across multiple dimensions **(**Table 2**)**, including TL measurement modality, biological specimen type, study design and covariate adjustment. This variability persisted even after assay-specific stratification, where studies using the same measurement approach differed in sample source, analytical methods, and confounder control. Consequently, the findings were presented narratively. Most studies employed a cross-sectional design (*n* = 37), followed by longitudinal studies (*n* = 13), mixed cross-sectional/longitudinal designs (*n* = 6), prospective cohort/cross-sectional (*n* = 1), retrospective cohort/longitudinal (*n* = 1), and retrospective cohort designs (*n* = 1). TL was assessed using three main modalities: qPCR (*n* = 44), TRF analysis (*n* = 9), and DNAmTL (*n* = 6). Biological specimens and cell compartments varied widely, including peripheral blood mononuclear cells (PBMCs, *n* = 33), whole blood (*n* = 19), peripheral blood leukocytes (PBLs, *n* = 3), isolated naïve and memory CD8 T cells (*n* = 1), lymphocyte subsets (*n* = 1), white blood cells (*n* = 1), and serum (*n* = 1). Adjustment for key confounders during TL analysis was inconsistent; age was accounted for in 45 studies, smoking in 27, HCV status in 15, ART duration in 12, and viral load in 17.

**Table 1:** Summary of all included studies related to telomere length dynamics in PLWH

Reference	Aim of study	Study design/ Population	Telomere method	Main findings
[34]	To characterize immune senescence across T cell subsets in suppressed Chinese Men who have sex with men (MSM) and ART-naïve controls	Cross-sectional, 56 MSM	qPCR	Memory CD8 T cells had significantly shorter telomeres than naïve CD8 T cells in both ART groups (*P* < 0.001). Naïve and memory CD8 T cells in the ART group had slightly longer telomeres than cells in the ART-naïve group but this did not reach statistical significance (*P* > 0.05)
[35]	To compare the immune age of slow progressors (SP), virally suppressed (VL-), unsuppressed (VL+) and HIV-negative participants through quantifying cellular immune ageing markers in lymphocyte subsets	Cross-sectional, 198 participants	qPCR	SP and VL+ participants had similar immune profiles and shorter telomeres in their B cells, CD4 T cells and proliferative CD8 T cells compared to VL- and HIV- controls. VL- TL was not significantly different to HIV- TL. Elite controllers had similar ageing profiles to non-elite slow progressors
[36]	To determine the seroprevalence of seven latent/chronic viruses in HIV-positive and HIV-negative participants, assess the effects of sex and HIV status on viral infection risk, and evaluate associations between these infections and leukocyte TL	Cross-sectional, 376 participants	qPCR	In females, TL declined at a similar rate in both HIV- and HIV+ groups but PLWH had a lower y-intercept. TL in HIV+ women was significantly shorter than HIV- women (8.1 [7.8–8.3] vs. 8.8 [8.5–9.1], *P* < 0.001). In males, there was no significant difference between TL in HIV + and HIV- males (7.9 [7.5–8.3] vs. 8.3 [7.9–8.7], *P* = 0.02). Having 3–4 non-HIV infection associated with shorter leukocyte TL
[37]	To compare DNA methylation-based biomarkers of ageing among women with and without HIV and evaluate associations of epigenetic age acceleration with bone mineral density and physical function	Cross-sectional, 190 women	DNAmTL	Women with HIV had significantly shorter DNAmTL (7.12 ± 0.31 vs. 7.34 ± 0.23, *P* < 0.001) and significantly higher epigenetic age acceleration (mean ± SD, 1.44 ± 5.36 vs. -1.88 ± 5.07, *P* < 0.001)
[38]	To examine whether HIV suppression and improved CD4 counts after HAART initiation were associated with the degree of epigenetic acceleration	Longitudinal, 399 men	DNAmTL	Epigenetic age acceleration decreased after HAART initiation but remained accelerated when compared to the matched HIV- group. aaDNAmTL significantly increased intra-individually in PLWH from pre to post HAART (*P* < 0.001). A higher Post HAART cumulative viral load was associated with shorter aaDNAmTL (0.08 relative unit decrease per 10-fold increase). A higher lifetime cumulative viral load was associated with shorter aaDNAmTL (-0.11 relative unit decrease per 10-fold increase)
[39]	To assess ageing biomarkers and their correlation with the HIV reservoir in adolescents and young adults with perinatally acquired HIV (PHIVAYA)	Cross-sectional, 78 participants	qPCR	PHIVAYA had significantly shorter telomeres than healthy controls. The virally suppressed group had significantly longer telomeres than the unsuppressed group (1.2 [1.1–1.3] vs. 1.1 [1.1–1.2], *P* < 0.012). The early suppressor subgroup had significantly longer telomeres than the late suppressor subgroup (1.3[1.2–1.4] vs. 1.2[1.1–1.3], *P* < 0.143)
[40]	To investigate whether TL shortening is accelerated in a South African population co-infected with HIV and Helminths compared to those singly infected. Additionally, to compare TL data to participant’s biochemical and full blood count parameters	Cross-sectional, 200 participants	qPCR	The control group had significantly higher relative TL compared to the HIV+ (*P* = 0.0121), helminth-infected (*P* < 0.0001) and co-infected (*P* = 0.0042) groups. The helminth-infected (β= -0.62, *P* = 0.000) and HIV and helminth co-infected (β= -0.58, *P* = 0.031) groups had significantly shorter relative TLs compared to the uninfected control group when adjusting for full blood count and socio-demographic parameters
[41]	To examine the association between DNAmTL, cancer prevalence and mortality risk among people with and without HIV in the Veterans ageing cohort study (VACS) and the Women’s interagency HIV study Cohort (WIHS)	Cross-sectional, VACS = 1917 WIHS = 481	DNAmTL	PLWH had significantly shorter DNAmTL compared to negative controls (Regression line was lower on the y-axis, intercepts for PLWH and negative controls; 7.87 and 8.31 in VACS1, and 7.76 and 8.18 in WIHS, respectively). There was a significant negative association between DNAmTL and cancer for the model without age (*P* = 1.37E­ 04). DNAmTL was associated with a 40% increase in mortality risk (hazard ratio: 0.06 (0.44, 0.82), *P* = 1.43E-03)
[42]	To evaluate TL recovery in long-term suppressed PLWH over 6 years and compare their blood TL to HIV-negative peers of similar and older age	Prospective cohort/ Cross-sectional, 384 participants	qPCR	PLWH had significantly shorter TL than age and sex-matched blood donors (T/S, 1.07 [IQR, 0.95–1.17] vs. 1.28 [IQR, 1.12–1.48], *P* < 0.001) but longer telomeres than the elderly population (T/S, 0.89 [IQR, 0.77–0.98], *P* < 0.001). The median blood TL of PLWH increased by 2.9% after 6 years (+ 0.48% per year, T/S 1.04[ IQR, 0,93 − 1,16] at baseline vs. 1.07 [IQR, 0.95–1.17] at 6 years, *P* > 0.002)
[43]	To measure TL in leukocytes of PLWH, evaluate the distribution of TERT genotypes among these patients and the relationship between TL and specific genotypes	Cross-sectional, 176 men	qPCR	cART regimen and duration did not significantly affect relative TL. The TERT rs273698 GG + AA genotype had significantly shorter telomeres (*P* = 0.049) than the GA genotype
[44]	To understand ovarian ageing in women living with HIV by comparing anti-Mullerian hormone (AMH) levels and predictors of AMH between infected and uninfected groups and examine its association with leukocyte TL	Cross-sectional/ Longitudinal, 462 women	qPCR	Women with HIV had shorter telomeres (7.2 vs. 7.5) and lover AMH (1.5 vs. 2.3 ng/ml) than controls. After adjusting for other variables, HIV was linked to 20% lower AMH levels in women under the age of 35, while shorter leukocyte TL was associated with AMH levels less than 2 ng/mL in women over the age of 35
[45]	To estimate the rate of biological ageing in PLWH of different chronological age under different therapy regimens	Cross-sectional, 205 men	qPCR	Men with HIV had longer telomeres than negative controls, although this did not reach significance. In the HIV+ group, TL of younger patients did not differ significantly to that of older patients, while in the HIV- group, telomeres of younger participants were significantly longer than that of the older subjects (*P* < 0.026). Both plasma viral load and duration of therapy were also related to TL
[46]	To investigate whether simplifying treatment to a dual therapy (DT) of dolutegravir/lamivudine affects blood TL from baseline to 48 weeks, compared to a standard triple therapy (TT) containing an anchor drug and two nucleoside reverse transcriptase inhibitors (NRTIs)	Longitudinal, 120 participants	qPCR	An increase in blood TL was observed at week 48. Within group analysis showed that the DT group had a significant gain in TL (+ 0.161 [95% CI 0.054–0.268] *P* = 0.004) while the TT group did not have a significant increase in TL (+ 0.011 [95% CI -0.041–0.065] *P* = 0.656). Participants who stopped abacavir showed a significant increase in mean TL at 48 weeks (+ 0.346 [95% CI 0.136–0.556] *P* = 0.003). Being female, younger and with a higher CD4/CD8 ratio was associated with higher baseline TL
[47]	To compare patients’ relative TL between and within different combined ART classes and to estimate the impact of certain HIV-related variables on relative TL	Cross-sectional, 176 men	qPCR	There was no significant difference in relative TL between the three therapy groups (*P* = 0.761). cART containing NNRTI had a significant impact on relative LT. Patients using efavirenz had significantly shorter telomeres (2.10 ± 1.55) when compared to patients taking nevirapine (3.33 ± 1.63) within combined ART (*P* = 0.018)
[48]	To examine the rate of epigenetic ageing in men living with HIV from shortly after seroconversion (within 2.5 years) to just before HAART initiation (< 1.5 years prior).	Longitudinal, 201 men	DNAmTL	Rate of epigenetic ageing in men with HIV was approximately twice as fast as that of uninfected men. Average rate of shortening in DNAmTL was significantly faster in HIV-infected men (-0.056 units per year of chronological age vs. -0,019, *P* < 0.0001), indicating a nearly 3-fold increase in rate of shortening compared to uninfected controls
[49]	To assess the rate of TL change over > 3 years of untreated chronic HIV infection, and to determine whether this change persists during > 3 years of suppressive ART in participants from the Swiss HIV Cohort Study	Longitudinal, 905 participants	qPCR	In women, baseline TL was 19.2% shorter than men (*P* = 0.002). Pre-ART, TL shortened significantly (annual change, -2.12% [95% CI, 6.9%−31.5%] *P* = 0.002) in men. On suppressive ART, there was no evidence of further telomere shortening (annual change, 0.54% [95% CI, -0.55%−1.63%] *P* = 0.33) in men and no evidence of any differences in TL change by sex
[50]	To understand the mechanism underlying the negative impact of tenofovir on TL in aviraemic PLWH by measuring TL and telomerase activity in whole blood, PBMCs CD4+ and CD8+ T cell populations	Cross-sectional, 128 participants	qPCR	Participants treated with tenofovir had significantly shorter median TL in PBMCs (*P* = 0.022) and CD8+ T cells (*P* = 0.003) in the crude analysis. No significant difference between treatment groups in whole-blood TL (*P* = 0.152) or CD4+ T cell TL (*P* = 0.232). After adjusting for demographic and clinical variables, only CD8+ T cell TL remained significantly shorter in tenofovir-exposed participants (*P* = 0.04). Age-related analyses further showed that tenofovir-treated participants had shorter telomeres across the entire age range than participants receiving tenofovir-sparing therapies
[51]	To investigate the impact of initial HIV infection on biological ageing by examining longitudinal changes in multiple DNA methylation–based measures of epigenetic age	Longitudinal, 204 MSM	DNAmTL	At visit B (following initial HIV infection) the seroconverters showed a significant difference in epigenetic age and shorter aaDNAmTL (all *P* < 0.001) compared to the matched seronegative group. From Visit A (before infection) to Visit B, aaDNAmTL shortened significantly in HIV+ men, with an estimated change of -0.264 relative units (*P* < 0.001), which was 17.6-fold greater than the estimated change of the seronegative group. A higher viral load at visit B was associated with shorter aaDNAmTL (*P* = 0.025)
[52]	To assess if HIV infection is associated with epigenetic age acceleration in African American adults ≥ 60 years by first and second-generation epigenetic clocks and to evaluate if epigenetic age acceleration is associated with frailty and cognitive function	Cross-sectional, 107 participants	DNAmTL	PLWH had higher epigenetic age acceleration (2.39 ± 8.5 vs. -4.34 ± 5.6, *P* < 0.001) and lower DNAmTL (6.77 ± 0.35 vs. 7.07 ± 0.20, *P* < 0.001) compared to negative controls. DNAmTL was significantly lower among PLWH with a detectable viral load ≥ 50 copies/ml and in those with a CD4 count < 200 cells/mm^3^
[53]	To evaluate the association between time of ART start and TL over a 6-year time period in participants with primary HIV infection in the Zurich primary HIV infection study (ZPHI) and to estimate the impact of early ART start relative to other factors known to be associated with TL	Longitudinal, 105 participants	qPCR	No difference in median TL from first to last sample in the ART continuous and ART interrupted groups (-1.21%/year vs. -1.55%/year, *P* = 0.55). Participants with a shorter ART delay had a longer median baseline TL (1.44 [IQR 1.18–1.69]). Participants who delayed ART for 60 days had 22.6% shorter than those that delayed for just 25 days
[54]	To examine the impact of HIV infection and methamphetamine use on leukocyte TL	Cross-sectional, 161 participants	qPCR	PLWH had shorter unadjusted T/S ratios than uninfected groups (*P* = 0.003). Multivariable analyses showed that only plasma CRP and cerebrospinal fluid sVCAM-1 were associated with T/S ratio (model R^2^ = 0.62, *P* < 0.0001). A lower T/S ratio was associated with an increased risk of cardiovascular disease (*P* < 0.0001) and stroke (*P* < 0.0001), weaker motor functioning (*P* = 0.024) and processing speed (*P* = 0.013), more depressed symptoms (*P* = 0.0083), and more CSF neurofilament-light (*P* < 0.0001)
[55]	To evaluate any independent association between TL and coronary artery disease (CAD) events in PLWH, in the context of all relevant clinical risk factors, HIV-related risk factors and adverse antiretroviral exposures	Cross-sectional/ Longitudinal, 1078 participants	qPCR	Relative TL from the first to last sample was similar in both cases and controls (-1.83% [IQR -3.69% to -3.43%] vs. -1.04% [IQR − 3.46% to 2.85%], *P* = 0.40). In the first sample, TL was associated with CAD, with an odds ratio (OR) per unit increase of 0.65 (95% CI: 0.48–0.88). In the last sample, TL was not associated with CAD, OR = 0.92 (95% CI, 0.72–1.18). TL decline from first to last sample was not associated with CAD
[56]	To investigate the relationship between TL and fat redistribution among participants with HIV	Cross-sectional, 27 men	qPCR	TL was reduced in men with HIV vs. HIV negative controls (5.05 ± 0.70 vs. 5.54 ± 0.61; *P* = 0.08). Iliac waist circumference (113.4 ± 10.0 vs. 102.7 ± 11.5 cm; *P* = 0.05), waist-to-hip ratio (1.06 ± 0.07 vs. 0.98 ± 0.09; *P* = 0.04), and visceral adipose tissue (301.5 ± 94.4 vs. 187.7 ± 69.6 cm2; *P* = 0.01) were significantly greater among participants with HIV having below-median TL compared to participants with HIV having above-median TL
[57]	To characterize the relationship between leukocyte TL and whole blood (WB) mitochondrial DNA (mtDNA) content in a cohort of girls and women living with and without HIV both cross sectionally and longitudinally	Cross-sectional/ Longitudinal, 908 women	qPCR	Cross sectionally, women with HIV had shorter TL (median 7.1 vs.7.4) and lower WB mtDNA content (median 101 vs. 112) than controls before (*P* ≤ 0.004) and after adjusting for age (*P* ≤ 0.002). Leukocyte TL was modulated by HIV status, whereby the decline in TL with age was faster among HIV+ women with detectable pVL compared to negative controls. HIV infection, current tobacco smoking, and older age were independently associated with shorter TL. A slower decrease in WB mtDNA content/year was a strong predictor of faster leukocyte TL loss
[58]	To examine the association between TL changes after 96 weeks of initial ART and changes in T cell subpopulations in a subgroup of participants from the NEAT001/ANRS 143 trial	Longitudinal, 31 participants	qPCR	Mean blood TL increased significantly from week 0 to week 96 (*P* = 0.03). Adjusted for baseline TL, TL was slightly higher in participants treated with DRV/r + RAL compared to DRV/r + TDV/FTC, but this difference was not significant (mean TL = 0.05, *P* = 0.12). TL change correlated positively with CD4 + T cell percentage (TL increase by 0.46 per 100% increase, *P* = 0.03) and negatively with CD4 + effector memory T cells (TL decrease by -0.55 per 100% increase, *P* = 0.01), with similar trends for effector and terminal effector memory CD4+ T cells
[59]	To investigate whether six genetic variants previously associated with Leukocyte TL in a cohort of Africans living with and without HIV and undergoing evaluation for tuberculosis	Cross-sectional, 434 participants	qPCR	Only one single nucleotide polymorphism (SNP) had a significant effect on leukocyte TL, with the allele A of OBFC1 (rs9420907) being associated with shorter leukocyte TL in unadjusted (β = −0.04, *P* = 0.024) and adjusted (β = -0.04, *P* = 0.017) analyses
[60]	To examine longitudinal leukocyte TL during the course of pregnancy in women living with HIV receiving combination ART and HIV-negative women	Longitudinal, 105 women	qPCR	A univariable analysis showed HIV+ status, history of HCV infection and smoking throughout pregnancy were significantly associated with shorter TL. There was a significant interaction between maternal age and gestational age on leukocyte TL (*P* = 0.002 overall and *P* = 0.02 in HIV+ women). Telomere lengthening was seen as pregnancy progressed in women younger that 35. Smoking throughout pregnancy was the second strongest predictor of shorter leukocyte TL among all women (*P* = 0.06). However, this association only reached statistical significance in women with HIV (*P* = 0.04)
[61]	To compare thymic output, T cell receptor repertoire and TL of perinatally HIV-infected youths (pHIVy), with almost three decades of infection, to those of age-matched non-perinatally infected youth (npHIVy) and Healthy controls (HC)	Cross-sectional, 80 participants	qPCR	Median TL in PBMCs was significantly lower in pHIVy and npHIVy, than in HC (*P* < 0.0001). No correlation between TL, age and gender was found. There was no association between TL and the ongoing antiretroviral regimen. A significant correlation was observed between the extent of TL and the number of CD4+ cells in the same group of patients (*P* = 0.0001)
[62]	To investigate leukocyte TL in combination ART treated PLWH who achieved viral control, to identify factors associated with shorter TL and examine the influence of Tl on leukoaraiosis	Cross-sectional, 472 participants	qPCR	Leukocyte TL was significantly shorter in PLWH compared to negative controls (*P* < 0.0001). Older age, past/present history of substance use (*P* = 0.0058), failure to achieve viral loads < 40 RNA copies/mL within 1 year of initiating cART (*P* = 0.0053), and cART without INSTI were significantly associated with shorter TL. White matter hypersensitivity (WMH) was detected in 36 out of the 184 PLWH and was significantly associated with older age (*P* < 0.0001), shorter Leukocyte TL (*P* = 0.0006), hypertension (*P* = 0.0128), and carotid artery plaque (*P* = 0.0374)
[63]	To evaluate the levels of systemic inflammation and understand the risk of age-associated diseases in PLWH on long-term suppressive ART using biomarkers of inflammation and immune activation	Cross-sectional, 137 participants	qPCR	HIV-1 positive status had a negative association with TL (*P* < 0.0001) with the ART group having significantly shorter telomeres. Within the ART group, CXCL1 and TGF-α had a significant association with increased TL (*P* = 0.048) and (*P* = 0.026). L10RA was significantly associated with decreased TL (*P* = 0.042)
[64]	To investigate factors associated with baseline blood TL in participants enrolled in NEAT 001/ANRS 143, a trial comparing ritonavir-boosted darunavir (DRV/r) plus raltegravir (RAL) with DRV/r plus tenofovir disoproxil fumarate/emtricitabine (TDF/FTC) in ART-naïve PLWH	Cross-sectional, 201 participants	qPCR	Univariate analysis showed an association between shorter TL and older age (*P* < 0.001), HIV-1 RNA ≥ 100 000 copies/mL (*P* = 0.001), baseline CD4 count < 200 cells/uL (*P* = 0.037), lower CD4:CD8 ratio (*P* = 0.018), statin treatment (*P* = 0.004) and current alcohol consumption (*P* = 0.035). Multivariable analysis showed a decrease in baseline TL with age, 8% attrition for every 10 years (*P* < 0.001). Participants with HIV RNA ≥ 100 000 copies/mL had baseline TLs 6% shorter than those with HIV RNA < 100 000 copies/mL (*P* = 0.054)
[65]	To evaluate blood TL changes in a sub study of the NEAT 001/ANRS 143 clinical trial that compared ritonavir-boosted darunavir (DRV/r) plus raltegravir (RAL) with DRV/r plus tenofovir disoproxil fumarate/emtricitabine (TDF/FTC) in ART-naïve adults	Longitudinal, 201 participants	qPCR	At week 96 both ART groups experienced a gain in TL, however the TDF/FTC exposed participants had a gain in mean TL that was 0.031 superior to the raltegravir-exposed participants (*P* = 0.009). Independent predictors of gain in TL were baseline TL (*P* < 0.001), treatment with TDF/FTC (*P* = 0.005), and no current alcohol consumption at baseline (*P* = 0.026)
[66]	To study blood TL changes in HIV-infected individuals who became virally suppressed during treatment with tenofovir disoproxil fumarate (TDF) containing or TDF sparing ART.	Cross-sectional/ Longitudinal, 172 participants	qPCR	At study entry it was observed that participants receiving TDF or any N(t)RTIs had significantly shorter TL than those on N(t)RTI-sparing regimens (*P* = 0.018 and *P* = 0.035 respectively). Mean TL increased in the whole cohort (*P* = 0.030). After 2 years, the non-TDF group had longer telomeres (*P* = 0.011) and participants on N(t)RTI-sparing regimens had significantly longer TL than those on TDF, N(t)RTI or NRTI containing regimens
[67]	To investigate novel biological variables associated with poor bone mineral density (BMD) in women living with HIV	Cross-sectional, 353 participants	qPCR	Leukocyte TL (*P* = 0.009) and BMI (*P* = 0.042) were the strongest predictors of Lumber spine BMD. The only variable showing a significant association with leukocyte TL was lifetime tenofovir (TDF) exposure, *P* = 0.04 (slope = -11.53 ± 5.5). Of the 73 women with HIV, 81% had been exposed to TDF and their mean leukocyte TL was 2.88 ± 0.5 while the TDF naive women (*n* = 14) had a mean leukocyte TL of 2.94 ± 0.5
[68]	To examine the association between insomnia symptoms and leukocyte TL and how this association is affected by HIV serostatus and age	Cross-sectional, 488 participants	qPCR	PLWH had a higher prevalence of insomnia symptoms than uninfected participants (18.9% vs. 9.4%, *P* = 0.032). PLWH had shorter leukocyte TL than uninfected participants (0.82 vs. 0.89, *P* = 0.052). Among the aged PLWH (55–82 years), those with insomnia symptoms had lower geometric means of leukocyte TL than those without insomnia symptoms (0.664 vs. 0.84; *P* unadjusted = 0.050 and *P* age-adjusted = 0.026)
[69]	To evaluate the association between cognitive deficits and leukocyte TL in HIV-1 infected individuals	Cross-sectional, 164 participants	qPCR	PLWH, both with and without neurocognitive disorder, had shorter TL compared to healthy controls (*P* = 0.0073). There was no significant difference in TL between HIV-positive individuals with or without neurocognitive disorders, suggesting that TL alone cannot distinguish between levels of cognitive impairment in PLWH. HIV+ patients without neurocognitive disorder had longer telomeres than those with cognitive impairment (*P* = 0.01)
[70]	To determine the impact of tenofovir (TFV) on the TL of PLWH receiving ART.	Cross-sectional, 200 participants	qPCR	Patients exposed to TDF had telomeres 4% shorter than those of patients who never received TDF, but this difference did not reach statistical significance (*P* = 0.238). TL decreased with time on N(t)RTI, with 4% attrition for every 5 years of treatment with N(t) RTIs (*P* = 0.01)
[71]	To identify when age acceleration might occur in HIV-infected injection drug users and describe key biologic pathways disturbed during the HIV seroconversion period	Longitudinal, 31 participants	qPCR	Mean TL (± SD) was 227 ± 46 kbp/genome at T1, 201 ± 48 kbp/genome at T2, and 186 ± 27 kbp/genome at T3. TL at T2 was significantly shorter than T1 (*P* = 0.045), but TL at T3 was not significantly different from T2 (*P* = 0.244). Epigenetic age acceleration positively correlated with days since HIV infection (*P* = 0.035)
[72]	To measure TL before and immediately after HIV and or hepatitis C virus (HCV) seroconversion and explore whether apparent telomere shortening occurs immediately after HIV/HCV seroconversion	Retrospective cohort, Longitudinal, 96 participants	qPCR	No differences in TL at T1 between participants in the 3 groups. At T2, TL was significantly shorter in both seroconverter groups (HCV: 8.4 [IQR 7.2–9.9] and HIV: 8.2 [IQR 6.9–10.0] vs. Controls: 9.6 [IQR 8.8–11.2], *P* = 0.02 and *P* = 0.01, respectively). TL was significantly shorter (− 13%) post seroconversion in HIV+ participants (8.2 [IQR 6.9–10.0] vs. 9.1 [IQR 7.8–11.1], *P* = 0.02), but not among HCV seroconverters (8.4 [IQR 7.2–9.9] vs. 8.5 [IQR 6.9–10.0], *P* = 0.55)
[73]	To analyze immune parameters indicative of chronic immune activation and immune senescence, and to compare these markers between HIV-infected individuals with virologic suppression on ART and uninfected controls	Cross-sectional, 189 participants	qPCR	PLWH on ART had significantly shorter telomeres than controls (*P* < 0.001). During ART, shorter telomeres were associated with higher levels of CD4+ activated T cells (*P* = 0.004) and higher levels of the monocyte activation markers sCD14 and sCD163 (*P* = 0.010)
[74]	To measure TL and examine whether short telomeres are associated with HIV infection, TB and mortality in a cohort of African patients with and without HIV that have pneumonia	Cross-sectional, 184 participants	qPCR	Age, gender, cigarette smoking history, alcohol consumption history, and asthma were all associated with TL. HIV infection was seen as an independent predictor of shorter telomeres (*P* = 0.02). No significant correlation was found between TL and a TB diagnosis (*P* = 0.39) and short-term mortality, whether in the hospital (*P* = 0.93) or 2 months after admission (*P* = 0.45)
[75]	To quantify the magnitude of absolute TL (aTL) shortening that occurs with HIV and to investigate whether measures of chronic obstructive pulmonary disease (COPD) severity are associated with TL in PLWH	Cross-sectional, 922 participants	qPCR	Older age (*P* = 0.026), lower BMI (*P* = 0.011) and prior marijuana smoking (*P* = 0.015) were significantly associated with shorter TL. HIV+ participants had significantly shorter telomeres than CanCOLD participants (123 ± 4 vs. 150 ± 3 kbp/genome, *P* < 0.0001). The slopes of aTL vs. age were similar between the two groups, with a common slope of -0.713 ± 0.155 kbp/genome/year (*P* < 0.001). Duration of HIV infection (*P* = 0.019) and lower nadir CD4 cell counts (*P* = 0.023) were associated with shorter aTL. A greater proportion of HIV+ participants with the shortest TL had sever emphysema (*P* = 0.049)
[76]	To investigate the relationship between TL and immune recovery 48 weeks after cART initiation, and to assess the impact of nitrosative stress on oxidative stress during HIV-1 immune recovery following ART	Retrospective cohort, 132 participants	qPCR	Increases in CD4+ T cell counts at 48 weeks from cART initiation were greater in patients with long TL than in those with medium and short TLs (*P* = 0.007) with mean increase of 266 (236 to 297) cells per milliliter vs. 189 (144 to 235) and 189 (114 to 265), respectively. After adjusting for sex, age, CD4+ T cell counts, VL, and HCV infection at cART initiation, differences in mean CD4 T cell count increases remained statistically significant (*P* = 0.02)
[77]	To investigate leukocyte TL in PLWH and uninfected adults and to assess factors associated with shorter TL	Cross-sectional, 395 participants	qPCR	Shorter leukocyte TL was associated with older age (*P* < 0.001), HIV infection (*P* = 0.04), active hepatitis C virus infection (*P* = 0.02) and smoking (*P* < 0.003). Having a peak HIV pVL > 100 000 copies/mL was the only significant HIV-specific leukocyte TL predictor, even after adjusting for age (*P* < 0.05)
[78]	To evaluate the relationship between TL and immune activation markers among a cohort of men with and without HIV	Cross-sectional, 143 men	qPCR	TL was significantly shorter in the HIV population compared with the controls (*P* = 0.04). Immune activation markers hsIL-6 (*P* = 0.01), lipopolysaccharide (*P* = 0.0004) and sCD163 (*P* = 0.0007) were significantly higher among the HIV cohort compared to the controls. In multivariate modeling, both sCD163 (*P* = 0.05) and HIV-serostatus (*P* = 0.06) were independent predictors of shorter TL
[79]	To determine whether nucleos(t)ide reverse transcriptase inhibitors (NRTI) contribute to an accelerated loss in TL in PLWH on ART	Longitudinal, 124 participants	qPCR	Older patients had significantly shorter TL (*P* = 0.006), while women had significantly longer TL (*P* = 0.026). No significant association between TL and the duration of prior NRTI treatment (*P* = 0.894) or the use of a PI versus NNRTI (*P* = 0.107). In multivariable analysis, TL was significantly longer in PBMCs from patients in the DRV/r monotherapy arm, compared to the DRV/*r* + 2NRTIs arm (*P* = 0.008)
[80]	To assess whether there is evidence that PLWH have advanced biological ageing compared to HIV-seronegative individuals by comparing TL and CDKN2A expression	Cross-sectional, 486 participants	qPCR	Mean TL was significantly shorter in PLWH than in negative controls (T/S ± SE: 0.91 ± 0.007 vs. 1.07 ± 0.008), *P* < 0.0001). TL decreased with chronological age in PLWH (*P* = 0.03). Mean CDKN2A expression was higher in PLWH than in controls (0.45 ± 0.02 vs. 0.36 ± 0.03, *P* = 0.003)
[81]	To evaluate the effects of HIV-infection and chronic stress associated with childhood trauma on TL and to investigate whether Leukocyte TL is a risk factor for neurocognitive impairments	Cross-sectional, 128 women	qPCR	Women with HIV had significantly shorter relative mean leukocyte TL (0.61) compared to controls (0.98) (*P* < 0.01). No significant independent effect of childhood trauma on relative leukocyte TL (*P* = 0.47) and no significant interaction effect between HIV status and childhood trauma on relative leukocyte TL (*P* = 0.19). HIV+ individuals scored significantly less in the Controlled Oral Word Association learning test compared to their HIV- counterparts
[82]	To determine the effects of commonly used Nucleos(t)ide reverse transcriptase inhibitors (NRTIs) on telomerase activity and TL in vitro in activated PBMCs and ex vivo in PBMCs from PLWH and negative controls	Cross-sectional, sample size unclear	qPCR	At a therapeutic dose of 0.3 µM, tenofovir was shown to be the most powerful inhibitor of telomerase activity and resulted in the highest shortening of TL in vitro. PLWH had shorter TL in PBMCs compared to uninfected controls using a linear regression analysis on HIV status and adjusted for age, but this did not reach statistical significance (*P* = 0.061)
[83]	To investigate whether the negative effects of HIV-1 infection and ageing on naïve CD4+ T Cell compartments are additive or interactive	Cross-sectional and Longitudinal, 47 and 20 men	qPCR	HIV infection was associated with significant telomere shortening within both subsets of naïve CD4+ T cells (younger: *P* = 0.0004 for CD31+, *P* = 0.0096 for CD31- and older: *P* < 0.0002 for both naive subsets)
[84]	To investigate the TL of isolated populations of T cells from a group of HIV-infected long term virus survivors	Cross-sectional, 21 participants	TRF	Data showed increased telomere shortening of the memory (CD45RO) T cell subset in comparison to the naive (CD45RA) T cell population. This difference was statistically significant for the control group (*P* < 0.05) but did not achieve statistical significance within the HIV-infected group
[85]	To investigate the proliferative history and thymic output of PBMCs from fast progressors (FP), slow/non-progressors (S/NP), and uninfected controls in the GRIV cohort	Cross-sectional, 170 participants	TRF	Mean TRF lengths were significantly shorter in S/NP (*n* = 93, 7.59 ± 0.11 kb) and FP (*n* = 42, 7.25 ± 0.15 kb) compared to controls (*n* = 35, 9.17 ± 0.19 kb), *P* < 0.001. Rates of mean TRF length shortening calculated for each group by cross-sectional analysis were controls = -45 ± 20 bp/yr, S/NP = -41 ± 14 bp/yr, FP = -29 ± 17 bp/yr
[86]	To analyze TL in blood cell populations as a measure of replicative history in PLWH	Cross-sectional, 120 participants	TRF	HIV infection resulted in a five-fold or more age acceleration of the immune system’s circulating PBMC component (*P* < 0.0001). Controls had telomeres that were 0.6, 1.1 and 1.5 kilobases longer than patients in the early, mid- and late stages of disease progression, respectively. Later stage patients showed an average TL similar to that seen in normal 75-year-old subjects despite having a mean age of 37 years
[87]	To determine whether CD4+ T-cell TL indicates increased turnover leading to proliferative exhaustion in HIV infection	Cross-sectional and Longitudinal, 24 and 8 participants	TRF	Cross-sectionally TRF length did not differ significantly between HIV-infected individuals and controls. Mean TL in naïve CD4+ T cells were 8.6 ± 1.1 kb in HIV+ vs. 9.0 ± 0.9 kb in controls and in memory CD4⁺ T cells 7.4 ± 0.8 kb vs. 7.5 ± 0.9 kb respectively. Longitudinally the eight HIV+ men showed normal telomere shortening rates in naïve (+ 6 ± 64 bp/year) and memory (-26.3 ± 89 bp/year) CD4+ T cells
[88]	To evaluate if replicative senescence in CD4+ T cells complicates immune reconstitution in advanced HIV-1 infection (CD4 < 200/mm^3^)	Cross-sectional,	TRF	Advanced HIV infection (CD4 < 200 cells/mm³) was associated with significantly shorter CD4 T cell TRF compared to controls (*P* < 0.01) and a higher proportion of short telomeres < 5 kb (*P* < 0.05). These differences were not observed in HIV-infected individuals with CD4 counts > 200 cells/mm³. Telomerase activity in CD4 T cells remained detectable and was not significantly different from controls
[89]	To examine TRF as an indicator of T cell replicative history and to correlate it with longitudinal changes in lymphocyte subpopulations following initiation of ART	Longitudinal, 18 men	TRF	6 of the 7 participants had increased mean T cell TRF after 6 to 12 months of ART. There was an average gain of 350 base pairs in patients treated with a PI. In almost all the 11 patients, there was an increase in CD8+ T cell TRF after instituting the combined ART (lamivudine, zidovudine and ritonavir). An increase in CD8 T cell TRF was evident within 12 weeks after initiation of therapy and then stabilized after 24 to 48 weeks of therapy. Mean TRF length in CD4+ T cells did not increase
[90]	To quantify the long-term dynamics of TL and the effect of HIV infection on lymphocyte turnover rates	Longitudinal, 6 participants	TRF	For the two slow progressors followed up for 14 years, TRF length shortened at a rate of 120 ± 10 bp/ year. The two uninfected controls followed up for 8 and 10 years had an average TRF shortening rate of 50 and 60 bp/year respectively, which was 2-fold lower than the slow progressors. CD8 T cell TRF length shortened slightly faster (140 ± 10 bp/year) than CD4 cells (100 ± 10 bp/year) in the slow progressors. In fast progressors, CD8 cell TRF length shortened even faster (240 ± 10 bp/year)
[91]	To investigate whether HIV infection leads to alterations in TL within T cell subpopulations, reflecting their replicative history and to assess whether the residual replicative potential of these cells is affected in infected individuals	Cross-sectional, 7 pairs of monozygotic twins	TRF	TRF length in CD4+ cells was significantly greater in the infected twins (mean difference 1.2 ± 0.4 kb). The opposite pattern was observed for TRF length in CD8+ T cells, which were shorter in HIV+ twins (mean difference 1.1 ± 0.3 kb). In the HIV+ twins TRF length was longer in CD4+ than in CD8+ cells (mean difference 0.9 ± 0.4 kb)
[92]	To evaluate whether expanded populations of non-proliferative CD28- CD8 + T cells in PLWH have shortened telomeres	Cross-sectional, 12 men	TRF	Slight but significant difference in mean TRF between the men with HIV and uninfected controls (*P* = 0.025). There was a higher proportion of CD8+ cells lacking expression of the CD28 antigen in men with HIV. TRF length of CD28- CD8+ cells was significantly shorter than that of CD28+ CD8+ cells (*P* = 10^− 4^). The mean TRF length of CD28-CD8+ cells of the men with HIV was significantly shorter than that of CD28-CD8+ cells of seronegative controls (*P* = 0.003)

Note: HAART (Highly active antiretroviral therapy), NNRTI (Non-nucleoside reverse transcriptase inhibitor), PBMC (Peripheral Blood Mononuclear Cell), aaDNmTL (age-adjusted DNAmTL), CRP (C-reactive protein), CSF (Cerebrospinal Fluid), cART (Combination ART), INSTI (Integrase strand transfer inhibitor), PI (Protease Inhibitor)

**Table 2:** Stratified summary of included studies

Study	Measurement modality	Study Design	Cell type	Age adjustment	Smoking adjustment	HCV status adjustment	ART duration adjusted	Viral load control adjusted
[34]	qPCR	Cross-sectional	Isolated naïve and memory CD8 T cells	No	No	No	No	No
[35]	qPCR	Cross-sectional	Lymphocyte subsets	Yes	Yes	Yes	No	No
[36]	qPCR	Cross-sectional	Whole blood	Yes	Yes	No	No	No
[37]	DNAmTL	Cross-sectional	PBMC	Yes	Yes	No	No	No
[38]	DNAmTL	Longitudinal	PBMC	Yes	Yes	No	No	No
[39]	qPCR	Cross-sectional	PBMC	Yes	No	No	Yes	No
[40]	qPCR	Cross-sectional	Whole blood	Yes	Yes	No	Yes	Yes
[41]	DNAmTL	Cross-sectional	Whole blood	Yes	Yes	No	No	No
[42]	qPCR	Prospective cohort/ Cross-sectional	Whole blood	Yes	No	No	No	No
[43]	qPCR	Cross-sectional	PBMC	Yes	No	No	Yes	No
[44]	qPCR	Cross-sectional/ Longitudinal	Whole blood	Yes	No	No	No	No
[45]	qPCR	Cross-sectional	Whole blood	No	No	No	No	No
[46]	qPCR	Longitudinal	Whole Blood	Yes	No	No	No	No
[47]	qPCR	Cross-sectional	PBMC	No	Yes	Yes	No	No
[48]	DNAmTL	Longitudinal	PBMC	Yes	Yes	No	No	Yes
[49]	qPCR	Longitudinal	PBMC	Yes	Yes	Yes	No	Yes
[50]	qPCR	Cross-sectional	PBMC	Yes	Yes	No	No	No
[51]	DNAmTL	Longitudinal	PBMC	No	Yes	No	No	No
[52]	DNAmTL	Cross-sectional	Whole blood	Yes	Yes	No	No	Yes
[53]	qPCR	Longitudinal	PBMC	Yes	Yes	Yes	No	No
[54]	qPCR	Cross-sectional	Whole blood	Yes	No	No	No	No
[55]	qPCR	Cross-sectional/ Longitudinal	PBMC	Yes	Yes	No	Yes	No
[56]	qPCR	Cross-sectional	PBMC	Yes	Yes	No	No	No
[57]	qPCR	Cross-sectional/ Longitudinal	Whole blood	Yes	Yes	Yes	No	Yes
[58]	qPCR	Longitudinal	PBMC	Yes	No	Yes	No	Yes
[59]	qPCR	Cross-sectional	PBMC	Yes	Yes	No	No	No
[60]	qPCR	Longitudinal	Whole blood	Yes	Yes	Yes	No	Yes
[61]	qPCR	Cross-sectional	PBMC	Yes	No	Yes	No	Yes
[62]	qPCR	Cross-sectional	PBLs	No	No	No	No	Yes
[63]	qPCR	Cross-sectional	PBMC	Yes	No	No	Yes	No
[64]	qPCR	Cross-sectional	Whole blood	Yes	Yes	Yes	No	Yes
[65]	qPCR	Longitudinal	Whole blood	Yes	Yes	Yes	No	Yes
[66]	qPCR	Cross-sectional/ Longitudinal	Whole blood	Yes	No	No	Yes	Yes
[67]	qPCR	Cross-sectional	Whole blood	Yes	Yes	Yes	Yes	Yes
[68]	qPCR	Cross-sectional	PBMC	Yes	Yes	No	Yes	Yes
[69]	qPCR	Cross-sectional	Whole blood	Yes	No	Yes	No	No
[70]	qPCR	Cross-sectional	Whole blood	Yes	Yes	Yes	Yes	No
[71]	qPCR	Longitudinal	PBMC	No	No	No	No	No
[72]	qPCR	Retrospective cohort/ Longitudinal	PBMC	Yes	No	No	No	No
[73]	qPCR	Cross-sectional	PBMC	Yes	No	No	No	No
[74]	qPCR	Cross-sectional	PBMC	Yes	Yes	No	No	No
[75]	qPCR	Cross-sectional	PBLs	Yes	Yes	No	No	No
[76]	qPCR	Retrospective cohort	White blood cells	Yes	No	Yes	No	Yes
[77]	qPCR	Cross-sectional	Whole blood	Yes	Yes	Yes	Yes	Yes
[78]	qPCR	Cross-sectional	Serum	Yes	Yes	No	No	No
[79]	qPCR	Longitudinal	PBMC	Yes	No	No	Yes	No
[80]	qPCR	Cross-sectional	PBLs	Yes	No	No	No	No
[81]	qPCR	Cross-sectional	Whole blood	Yes	No	No	Yes	Yes
[82]	qPCR	Cross-sectional	PBMC	Yes	No	No	No	No
[83]	qPCR	Cross-sectional/ Longitudinal	PBMC	Yes	No	No	No	No
[84]	TRF	Cross-sectional	PBMC	No	No	No	No	No
[85]	TRF	Cross-sectional	PBMC	Yes	No	No	No	No
[86]	TRF	Cross-sectional	PBMC	No	No	No	No	No
[87]	TRF	Cross-sectional/ Longitudinal	PBMC	No	No	No	No	No
[88]	TRF	Cross-sectional	PBMC	No	No	No	No	No
[89]	TRF	Longitudinal	PBMC	No	No	No	No	No
[90]	TRF	Longitudinal	PBMC	No	No	No	No	No
[91]	TRF	Cross-sectional	PBMC	No	No	No	No	No
[92]	TRF	Cross-sectional	PBMC	No	No	No	No	No

## Discussion

To our knowledge, this is the first systematic review to comprehensively summarize TL dynamics across the full spectrum of HIV infection, spanning diverse disease stages, treatment exposures, and clinical subgroups. Of the 59 included studies, the majority (62.7%) were cross-sectional, providing snapshots of TL at specific stages, while a smaller number (22%) were longitudinal, tracking how TL evolves within individuals. Despite significant heterogeneity in study populations and measurement techniques, ranging from qPCR to newer epigenetic estimates like DNAmTL, the collective evidence supports an association between HIV infection, shortened telomeres and biological ageing. However, while most studies report shorter TL in PLWH compared to the general population, the magnitude of this effect is highly variable and appears to be dependent on sex, ART status and the specific cell type analyzed.

A primary challenge in interpreting these findings is the lack of methodological uniformity regarding multivariable adjustment. Although chronological age was controlled for in 45 of the 59 studies, other critical determinants of telomere attrition were not consistently integrated; for instance, smoking was addressed in 27 studies, while HCV co-infection and ART duration were included in only 15 and 12 studies, respectively. Because these lifestyle factors and clinical stressors independently influence cellular senescence [25, 93, 94], their exclusion suggests that many reported associations may be subject to residual confounding and reverse causation. Consequently, while the observed trends are compelling, the inconsistent use of covariates may mask the independent effects of HIV on telomere shortening. This underscores the need for a more standardized approach to confounding in future research.

### HIV disease progression and telomere length

**Fig. 2:**
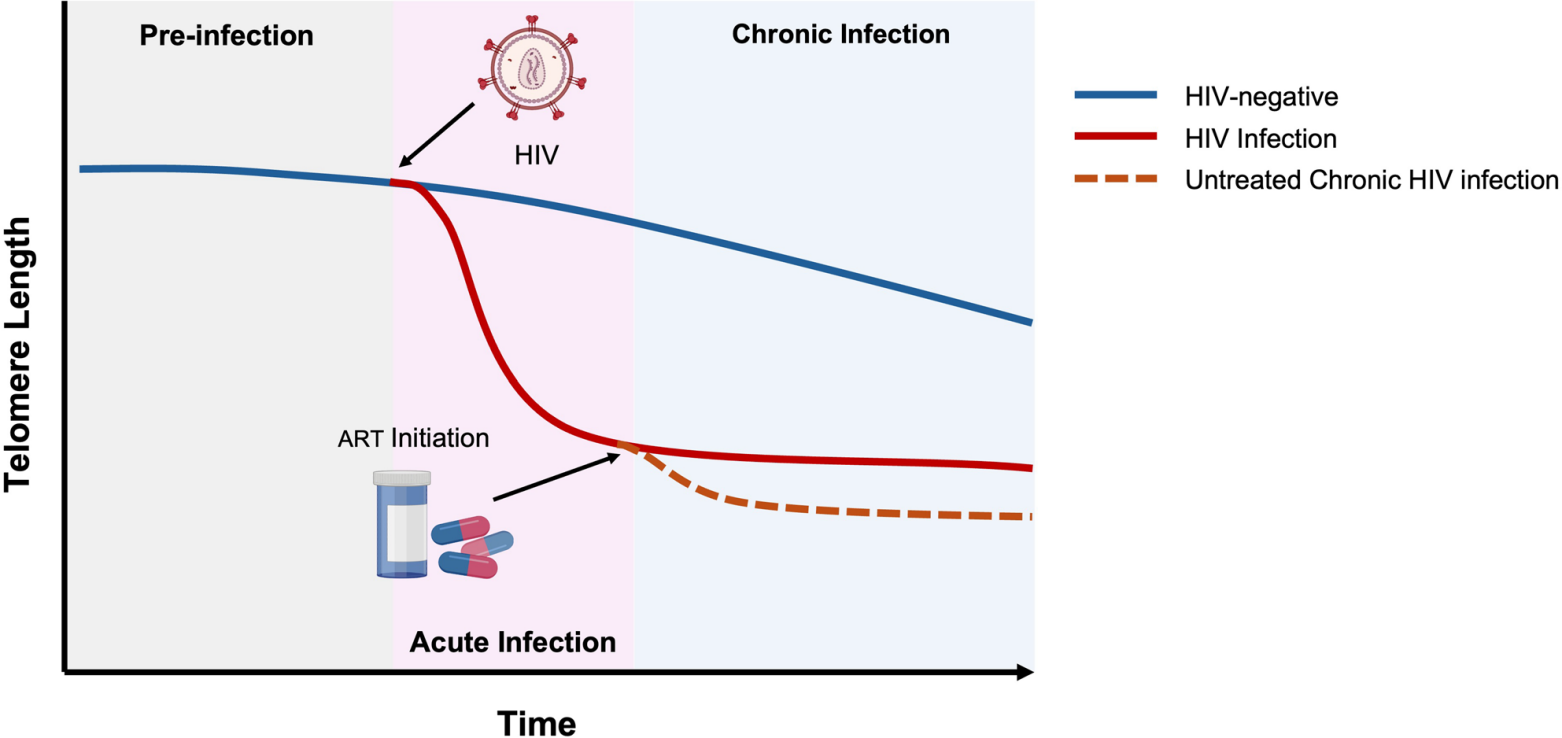
Changes in telomere length from pre-infection to chronic HIV infection

It appears that from the earliest moments of HIV acquisition, the foundation for premature telomere shortening is already laid. Liu et al., showed that in treated individuals, the age vs. TL relationship mirrors that of HIV-negative controls (a common slope of -0.713 ± 0.155 kbp/genome/year), suggesting that the steepest drop in TL occurs around the time of seroconversion [75]. This pattern was echoed across studies by Breen et al., and Gonzalez-Serna et al., Breen and colleagues reported significant DNAmTL shortening between samples collected prior to HIV infection (up to 1.5 years before the first seropositive visit) and after seroconversion (up to 2.5 years later), with the median estimated change being 17.6-fold greater than that observed in seronegative individuals. Gonzalez-Serna et al., reported that TL in samples collected a median of 9 months (IQR 8–14) after HIV seroconversion were 13% shorter than those pre-seroconversion [51, 72]. Leung et al., further demonstrated that TL declined significantly between pre-infection (277 ± 46 kbp/gemome) and early post-infection (201 ± 48 kbp/gemome) visits but then appeared to stabilize, with no meaningful additional shortening observed at subsequent time points (186 ± 27 kbp/gemome, *P* = 0.244) [71]. Biologically, viral dissemination during the acute stages of infection leads to aggressive immune activation and inflammation [95]. Immune cells proliferate in response to infection, resulting in a large population of cells with a history of replication and, consequently, shorter telomeres [7] as demonstrated in Fig. 2.

Following initial infection, subsequent telomere erosion is influenced by both the rate of disease progression and the duration of infection. Despite maintaining immune function for extended periods, slow progressors, including elite controllers, exhibit substantial telomere shortening, with TL and immune profiles similar to those of virally unsuppressed PLWH [35, 85, 86]. Fast progressors exhibit even more pronounced telomere attrition, with telomere loss rates reaching up to 240 bp/year [90]. Perinatally HIV-infected youths have also been reported to exhibit shorter telomeres compared to non-perinatally infected individuals. In a study by Petrara et al., the median relative TL was lower in perinatally infected youths (1.1 [IQR 1.1–1.2]) compared to non-perinatally infected individuals (1.2 [IQR 1.1–1.3], *P* = 0.012) [39]. However, Paghera et al., reported comparable telomere erosion rates between the two groups [61].

### Sex-specific patterns of telomere length in PLWH

In the general population, females are known to have longer telomeres than males [96, 97]. In contrast, most studies in PLWH did not find a significant sex-related difference in TL. However, many of these studies either included only one sex or had a disproportionately higher number of males, limiting the ability to make balanced comparisons. Interestingly, a longitudinal study observed that baseline TL in women was 19.2% (95% CI: 6.9%–31.5%) shorter than that of men pre-ART (*P* = 0.002), but the annual decline in TL did not differ by sex (difference in annual TL change in women vs. men = 0.02% [95% CI, -2.45% to 2.09%], *P* = 0.88) [49]. Yang et al., further noted that although the slope of TL shortening was steeper in males (*P* = 0.01), HIV-positive women had significantly shorter telomeres than HIV-negative controls (mean 8.1 [95% CI: 7.8–8.3] vs. 8.8 [95% CI: 8.5–9.1], *P* < 0.001). Conversely, the difference in TL between infected and uninfected males was less pronounced (mean 7.9 [95% CI: 7.5–8.3] vs. 8.3 [95% CI: 7.9–8.7], *P* = 0.20), suggesting that HIV may have a greater impact on TL in females. However, this study also reported a higher prevalence of co-infections among female participants with HIV, which could act as a confounder contributing to accelerated telomere shortening [36]. Nevertheless, another study reported that HIV-infected men experienced nearly a three-fold increase in epigenetic DNAmTL shortening (-0.056 units per year of chronological age vs. -0.019, *P* < 0.001) compared to negative controls [48], highlighting that both sexes are affected, potentially in unique ways.

Very few studies have explored TL in pregnant women living with HIV, likely because pregnancy itself can act as a significant confounder [98]. Most research in this population has focused on the TL of their infants. One study, however, reported a significant interaction between maternal age and TL in pregnant women with HIV (*P* = 0.02), with younger women (< 35 years) surprisingly showing telomere lengthening as pregnancy progressed. The authors suggested that this observation may reflect physiological changes during pregnancy, including increased blood volume that stimulates the production of new leukocytes with longer telomeres, as well as pregnancy-related differences in leukocyte turnover and hormonal regulation [60].

### ART-mediated modulation of telomere length

The relationship between ART and TL can be conceptualized as a biological balance between opposing mechanisms. On the one hand, ART suppresses viral replication, reducing immune activation and inflammation, which in theory, may allow partial telomere recovery by lessening replicative exhaustion. This pattern is reflected in studies examining epigenetic clocks in PLWH, where, following ART initiation, epigenetic age acceleration decreased [38]. Although PLWH continued to exhibit greater age acceleration than seronegative individuals, the between-group difference in median age-adjusted DNAmTL decreased from 0.53 to 0.39 relative units (≈ 26% reduction), suggesting partial normalization of epigenetic TL after ART [38]. On the other hand, certain ART classes, particularly Nucleoside reverse transcriptase inhibitors (NRTIs) such as tenofovir disoproxil fumarate (TDF), have been associated with delayed immune recovery [46, 50, 58, 66, 67, 70, 79] through the induction of oxidative stress and disruption of telomere elongation mechanisms.

### Cross-sectional associations between ART exposure and telomere length

Cross-sectional data consistently demonstrate that, despite viral control through ART, PLWH still possess shorter telomeres than their HIV-negative counterparts [63, 73, 99], but untreated PLWH experience the most substantial loss in TL. These findings highlight the enduring and largely irreversible damage HIV inflicts on the immune system. Both timing and the extent of viral suppression appear to be key drivers of these associations. Petrara et al., found that early suppressors (HIV-RNA plasma levels < 50 copies/ml achieved within 12 months of ART) maintained significantly longer telomeres compared to late suppressors (median 1.3 [IQR 1.2–1.4] vs. 1.2 [IQR 1.1–1.3], *P* = 0.012) [39], while Minami et al., noted that failing to achieve a viral load < 40 copies/mL within the first year of cART was a significant independent predictor of shorter leukocyte TL (*P* = 0.0053) [62].

Beyond viral suppression, several studies have explored whether specific antiretroviral drug classes are associated with differences in TL among PLWH. In a cross-sectional analysis comparing four major regimen classes (NNRTI-, INSTI-, PI-, and NRTI-based therapies), Bukic et al., reported no significant differences in relative TL across the treatment groups. However, NNRTI-containing regimens demonstrated the strongest association with relative TL (*P* = 0.018) [47]. Despite this, within the NRTI group, individuals receiving tenofovir/emtricitabine had the shortest mean relative TL, although this did not reach statistical significance. A similar pattern was observed by Montejano et al., who reported that participants exposed to TDF had telomeres approximately 4% shorter than those of TDF-naïve participants, though this difference was likewise not statistically significant [70]. While these findings should be interpreted cautiously, given the cross-sectional design, experimental evidence provides a potential biological explanation. In vitro work by Leeansyah et al., demonstrated that tenofovir, at therapeutic concentrations (0.3 µM), was a potent inhibitor of telomerase, the enzyme responsible for telomere elongation [82]. During telomere elongation, the reverse transcriptase subunit of telomerase adds nucleotide bases to telomeric DNA using an RNA template [100]. NRTIs act as structural analogues of natural nucleotides but lack the 3′-hydroxyl group required for phosphodiester bond formation [99]. While this mechanism primarily inhibits HIV reverse transcription, the structural similarity between HIV reverse transcriptase and telomerase reverse transcriptase [101] means that incorporation of NRTI analogues into telomeric DNA may similarly terminate chain elongation, thereby impairing telomere extension.

### Longitudinal telomere length dynamics following ART initiation

Longitudinal studies provide insight into changes in TL over time after ART initiation. Several cohorts have reported a modest gain in TL following treatment initiation [46, 65, 66, 89]. In one cohort comparing dual therapy (dolutegravir/lamivudine) with standard triple therapy, authors observed an overall increase in TL over 48 weeks, although this was only significant in the dual-therapy group (+ 0.161 [95% CI: 0.054–0.268], *P* = 0.004) while the triple-therapy group showed minimal change [46]. Similarly, in the NEAT001/ANRS 143 trial, which compared darunavir/ritonavir plus raltegravir with darunavir/ritonavir plus tenofovir/emtricitabine, mean TL increased from baseline to week 96, however, findings regarding regimen-specific differences were inconsistent. A sub-study reported significantly greater TL gains only in participants receiving darunavir/ritonavir plus tenofovir/emtricitabine (mean 0.031 [95% CI: 0.008–0.054]), more than the other group (*P* = 0.009) [65]. Conversely, later analysis of a smaller subgroup within the same clinical trial observed slightly higher gains in the raltegravir arm, although these differences were not statistically significant (*P* = 0.1186) [58], likely due to the smaller sample size.

Other studies suggest that ART primarily stabilizes telomere attrition rather than fully restoring TL. In a longitudinal cohort, telomeres shortened significantly prior to ART initiation (-2.12% annually, *P* = 0.002) but showed no further decline after viral suppression was achieved (annual change 0.54%, *P* = 0.33), indicating stabilization of telomere dynamics during treatment [49]. Similarly, Raffenberg et al., reported no significant difference in telomere change between continuous and interrupted ART groups (-1.21% vs. -1.55% per year, *P* = 0.55), although earlier treatment initiation was associated with longer baseline telomeres. Notably, delaying ART initiation by approximately two months was associated with telomeres that were 22.6% shorter than those of individuals who initiated treatment within 25 days [53].

Apparent gains in TL following ART initiation may reflect underlying biological changes in the immune system rather than true molecular elongation. By reducing chronic immune activation, ART shifts the circulating leukocyte pool from highly differentiated, short-telomere effector cells toward less differentiated naive and central memory cells, which have longer telomeres [8]. Supporting this mechanism, Babu et al., found that within ART-treated groups, levels of specific inflammatory mediators, including CXCL1 and TGF-α, were significantly associated with increased TL (*P* = 0.048 and *P* = 0.026) [63]. Consistent with these observations, Petrara et al., reported that early viral suppressors exhibited lower levels of immune activation, senescence, and exhaustion, alongside higher regulatory T and B cell levels, greater thymic output, and longer telomeres compared to late and non-suppressor groups, suggesting that immune restoration contributes to apparent telomere gains. Nevertheless, this recovery is never complete; PLWH often maintain elevated monocyte activation markers (sCD14 and sCD163), which are negatively correlated with TL [63, 73]. Importantly, these longitudinal “gains” must be interpreted with caution. They may represent apparent rather than true telomere restoration, driven by immune decompression, regression to the mean in qPCR-based assays, and survivor bias.

### Heterogeneity of telomere length in cells and HIV specific variables

**Fig. 3:**
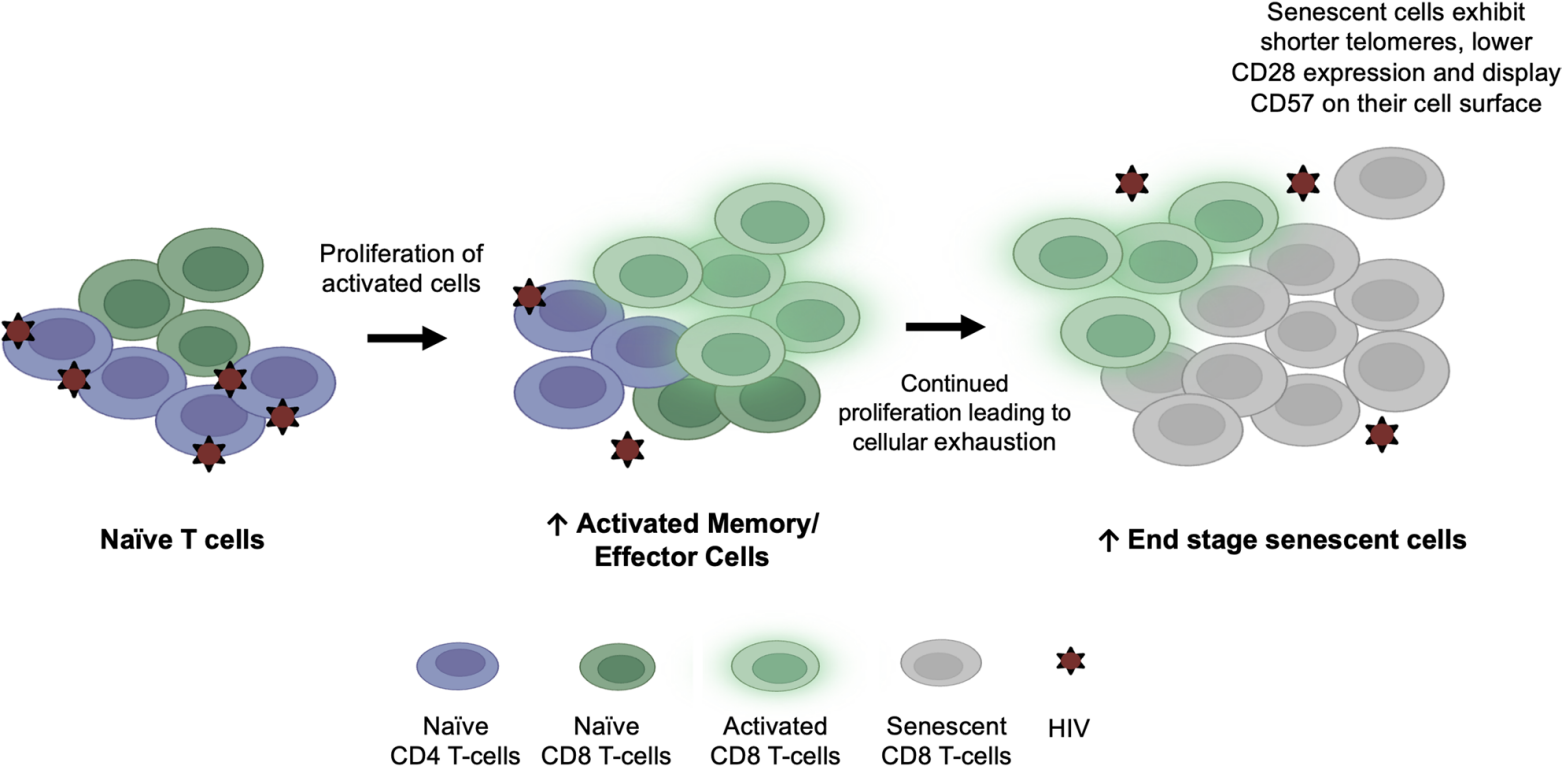
HIV-infection results in a shift from naïve to exhausted T cell subsets with significantly shorter telomeres

HIV disrupts normal T cell homeostasis, shifting the T cell compartment toward highly differentiated, antigen-experienced, and senescent phenotypes **(**Fig. 3**)** [10, 102]. Across the included literature, immune cell subsets display predictable telomere dynamics. Memory T cells exhibit shorter telomeres than their naïve counterparts [34, 58, 84], with CD8 T cells showing the greatest degree of telomere erosion [91]. Chalouni et al., demonstrated that longitudinal changes in TL corelated positively with CD4 T cell percentage (TL increase of 0.46 per 100% increase, *P* = 0.03) and negatively with the proportion of effector memory CD4 T cells (TL decrease of -0.55 per 100% increase, *P* = 0.01), with similar trends observed for effector and terminal effector memory CD4 T cells [58]. HIV-infected individuals also appear to have an increased proportion of CD8⁺CD28⁻ T cells, a population associated with cellular exhaustion, and these cells likewise possess significantly shorter telomeres [91].

In some studies, viral load (*P* = 0.0053) [62], CD4 count (*r* = 0.75, *P* = 0.0001) [61] and CD4/CD8 ratios (*P* = 0.018) [64] were significantly associated with shorter TL, whereas in others these associations did not reach significance, but differences in study population and method of TL measurement should be considered. Alejos et al., among other studies, demonstrated in their univariate analysis that both a HIV-1 RNA ≥ 100 000 copies/ml (*P* = 0.001) and a baseline CD4 count less than 200 cells/ul (*P* = 0.004) were linked to shorter telomeres [52, 64, 77]. Two studies have explored how genetic variation influences TL trajectories in PLWH. Telomerase is known to facilitate the elongation of telomeres. By studying single-nucleotide polymorphisms in the reverse transcriptase (TERT) component of telomerase, Bukic et al., found that TERT rs2736098 heterozygotes (GA) had significantly longer telomeres [43]. In an African cohort, Wang et al., observed a higher frequency of alleles associated with longer TL, but when evaluated individually, the allele A of OBFC1 (rs9420907) had a significant effect on TL, being associated with shorter telomeres [59]. Together, these findings indicate that host genetic factors may contribute to inter-individual differences in TL among PLWH, independent of HIV infection or treatment status.

### Telomere length and age-associated diseases in PLWH

Alongside its ability to reflect biological age, TL has been inextricably linked to clinically relevant outcomes. Evidence from this review confirms that HIV-related telomere shortening may predispose PLWH to a spectrum of age-associated medical conditions, including neurocognitive impairments [52, 69, 81], cardiometabolic diseases [54, 55], and malignancy. Findings from large, well-characterized cohorts such as VACS and WIHS demonstrated that shorter epigenetic DNAmTL, was associated with higher cancer prevalence and mortality. Specifically, after adjusting for relevant covariates, lower DNAmTL was associated with increased mortality risk, with each one-kilobase decrease corresponding to a 40% higher risk of death (HR = 0.60, 95% CI: 0.44–0.82) [41]. Similarly, lower T/S ratios were linked not only to cardiovascular disease and stroke but also to slower motor and processing speed performance [54], whereas longer baseline telomeres reduced the odds of developing coronary artery disease by almost 50% [55]. Even so, in the absence of studies reporting actual clinical endpoints, these findings should be interpreted as associations rather than evidence of direct causation. Shortened telomeres have also been associated with clinically relevant phenotypes such as abnormal fat redistribution among PLWH. Individuals with shorter telomeres exhibited higher waist circumference, increased waist-to-hip ratio, and substantially greater visceral adipose tissue compared to those with above median-TL [56]. While body fat is not an age-related anomaly per se, it is a clinically important phenotype known to influence age-associated disease risk [103]. Among women with HIV, telomere shortening has been associated with markers of accelerated ovarian ageing (lower levels of anti-Mullerian hormone) and earlier menopause [44], a shift that leads to greater vulnerability to type 2 diabetes, osteoporosis [67] and other diseases related to advancing age.

Given the links between TL and multiple age-related diseases, measuring this marker may help identify individuals at greater risk of specific health complications, allowing for early intervention. This later opens avenues for research into strategies to preserve or restore TL, with the goal of enhancing the health and quality of life of PLWH. Importantly, measurement methods such as DNAmTL reflect epigenetically inferred TL and capture cumulative ageing signatures distinct from direct leukocyte telomere measurements via qPCR. Therefore, these modalities should not be interpreted interchangeably.

### GRADE quality assessment

Nearly all the included literature was observational, primarily consisting of cross-sectional and longitudinal designs. While this is considered appropriate when looking at TL and HIV-related outcomes, the lack of randomization increases susceptibility to confounding and selection bias. Combined with inconsistencies in TL measurement methods and variability in study populations, the overall quality of evidence was rated as very low according to the GRADE assessment tool [33]. Similarly, Tunnicliffe et al., [31] rated the existing literature on HIV and TL as very low quality; however, 79% of the assessed HIV-related exposure-outcome relationships showed an effect on TL and remained robust in both adjusted and unadjusted analyses [31]. Consistent with this, the present review found that HIV serostatus repeatedly emerged as one of the strongest catalysts of telomere shortening. Taken together, these findings reinforce the notion that HIV infection influences biological ageing, with TL serving as a key measurable indicator of this effect.

### Future research priorities

Future research should prioritize well-designed longitudinal studies that incorporate repeated TL measurements to better understand changes over time and the factors driving these changes in PLWH. Greater harmonization of TL measurement approaches is needed to enhance inter-study comparability. In addition, future work should incorporate cell subset-specific analyses, ensure consistent adjustment for key confounders (e.g., age, smoking, HCV co-infection, ART exposure, and virological suppression), and prioritize mechanistic studies to define pathways linking persistent immune activation, inflammation, mitochondrial dysfunction, and epigenetic ageing to telomere dynamics. Such approaches will be critical for clarifying whether telomere shortening is primarily a biomarker of cumulative HIV-associated biological stress or a mechanistically relevant contributor to premature ageing phenotypes in this population.

### Limitations

Most included studies were cross-sectional, limiting insight into within-person telomere dynamics and preventing causal inference. There was considerable heterogeneity in study populations, telomere measurement methods, and clinical subgroup definitions, making direct comparisons and meta-analysis challenging. Sample sizes were often small, and confounders were inconsistently adjusted for, which may affect the reliability of findings. Given that PLWH were the population of interest, for individuals born to HIV-infected mothers who themselves remained HIV-uninfected, we cannot account for whether exposure alone to HIV in utero may affect TL. Finally, publication bias towards significant correlations, cannot be ruled out, as such studies reporting these associations are more likely to be published.

## Conclusions

Across the methodologically heterogeneous literature, HIV infection was frequently associated with shorter TL or accelerated telomere attrition, however, the magnitude and independence of this association varied according to assay platform, cell type, study design, and covariate adjustment. Despite these limitations, ART, particularly when initiated early and maintained, has been linked to slower telomere attrition and may support partial recovery. Both timing and regimen choice appear to influence the extent of this recovery. Host and clinical factors, including gender, viral load, CD4 count, and genetic variation further contribute to TL variability among PLWH. Taken together, these findings position TL as a biologically informative marker of ageing and cumulative HIV-related immune stress. While this supports its relevance in understanding ageing-related vulnerability in PLWH, the absence of standardized thresholds, consistent measurement approaches and robust longitudinal validation currently limits clinical translation. Addressing these gaps will require well-designed, long-term studies with repeated follow-up visits and clearly defined timepoints to accurately characterize telomere trajectories across the course of HIV infection and treatment.

## Supplementary Information

Below is the link to the electronic supplementary material.

Supplementary Material 1

## Data Availability

All data generated or analyzed during this study are included in this published article and its supplementary information files.

## Abbreviations

aaDNAmTL: Age-adjusted methylation-based telomere length
aTL: Absolute telomere length
AMH: Anti-Müllerian hormone
ART: Antiretroviral therapy
BMD: Bone mineral density
cART: Combination antiretroviral therapy
CAD: Coronary artery disease
COPD: Chronic obstructive pulmonary disease
CRP: C-reactive protein
CSF: Cerebrospinal fluid
DNAmTL: DNA methylation-based telomere length
DRV/r: Ritonavir-boosted darunavir
DT: Dual therapy
FP: Fast progressors
GRADE: Grading of Recommendations, Assessment, Development and Evaluations
HAART: Highly active antiretroviral therapy
HC: Healthy controls
HCV: Hepatitis C virus
INSTI: Integrase strand transfer inhibitor
MSM: Men who have sex with men
mtDNA: Mitochondrial DNA
NNRTI: Non-nucleoside reverse transcriptase inhibitor
npHIVy: Non-perinatally HIV-infected youths
NRTIs: Nucleoside reverse transcriptase inhibitors
PBMC: Peripheral blood mononuclear cell
PHIVAYA: Adolescents and young adults with perinatally acquired HIV
pHIVy: Perinatally HIV-infected youths
PI: Protease inhibitor
PLWH: People living with HIV
PRISMA: Preferred Reporting Items for Systematic Reviews and Meta-Analyses
qPCR: Quantitative polymerase chain reaction
RAL: Raltegravir
S/NP: Slow/ non-progressors
TDF/FTC: Tenofovir disoproxil fumarate/emtricitabine
TFV: Tenofovir
TL: Telomere length
TRF: Terminal restriction fragment
TT: Triple therapy
VL: Virally suppressed
VL+: Virally unsuppressed
WB: Whole blood

## References

[1] Deeks SG. Immune dysfunction, inflammation, and accelerated aging in patients on antiretroviral therapy. Top HIV Med. 2009;17(4):118–23. 19890183

[2] Lee KA, Flores RR, Jang IH, Saathoff A, Robbins PD. Immune Senescence, Immunosenescence and Aging. Front Aging. 2022;3:900028. 10.3389/fragi.2022.900028. 35821850 10.3389/fragi.2022.900028 PMC9261375

[3] Pawelec G. Age and immunity: What is immunosenescence? Exp Gerontol. 2018;105:4–9. 10.1016/j.exger.2017.10.024. 29111233 10.1016/j.exger.2017.10.024

[4] Thomas R, Wang W, Su DM. Contributions of Age-Related Thymic Involution to Immunosenescence and Inflammaging. Immun Ageing. 2020;17:2. 10.1186/s12979-020-0173-8. 31988649 10.1186/s12979-020-0173-8 PMC6971920

[5] Franceschi C, Garagnani P, Parini P, Giuliani C, Santoro A. Inflammaging: a new immune-metabolic viewpoint for age-related diseases. Nat Rev Endocrinol. 2018;14(10):576–90. 10.1038/s41574-018-0059-4. 30046148 10.1038/s41574-018-0059-4

[6] Fasching CL. Telomere length measurement as a clinical biomarker of aging and disease. Crit Rev Clin Lab Sci. 2018;55(7):443–65. 10.1080/10408363.2018.1504274. 30265166 10.1080/10408363.2018.1504274

[7] Appay V, Sauce D. Assessing immune aging in HIV-infected patients. Virulence. 2017;8(5):529–38. 10.1080/21505594.2016.1195536. 27310730 10.1080/21505594.2016.1195536 PMC5538339

[8] Dalzini A, Petrara MR, Ballin G, Zanchetta M, Giaquinto C, De Rossi A. (2020). Biological aging and immune senescence in children with perinatally acquired HIV. J Immunol Res. 2020:8041616. 10.1155/2020/8041616. 10.1155/2020/8041616 PMC7246406 32509884

[9] Desai S, Landay A. Early immune senescence in HIV disease. Curr HIV/AIDS Rep. 2010;7(1):4–10. 10.1007/s11904-009-0038-4. 20425052 10.1007/s11904-009-0038-4 PMC3739442

[10] Sokoya T, Steel HC, Nieuwoudt M, Rossouw TM. HIV as a Cause of Immune Activation and Immunosenescence. Mediators Inflamm. 2017;2017:6825493. 10.1155/2017/6825493. 10.1155/2017/6825493 PMC5676471 29209103

[11] Biver E. Osteoporosis and HIV Infection. Calcif Tissue Int. 2022;110(5):624–40. 10.1007/s00223-022-00946-4. 35098324 10.1007/s00223-022-00946-4 PMC9013331

[12] Carbone A, Vaccher E, Gloghini A. Hematologic cancers in individuals infected by HIV. Blood. 2022;139(7):995–1012. 10.1182/blood.2020005469. 34469512 10.1182/blood.2020005469

[13] Ntsekhe M, Baker JV. Cardiovascular Disease Among Persons Living With HIV: New Insights Into Pathogenesis and Clinical Manifestations in a Global Context. Circulation. 2023;147(1):83–100. 10.1161/CIRCULATIONAHA.122.057443. 36576956 10.1161/CIRCULATIONAHA.122.057443

[14] Watkins CC, Treisman GJ. Cognitive impairment in patients with AIDS - prevalence and severity. HIV AIDS (Auckl). 2015;7:35–47. 10.2147/hiv.S39665. 25678819 10.2147/HIV.S39665 PMC4319681

[15] Hamczyk MR, Nevado RM, Barettino A, Fuster V, Andrés V. Biol Versus Chronological Aging JACC. 2020;75(8):919–30. 10.1016/j.jacc.2019.11.062. 10.1016/j.jacc.2019.11.062 32130928

[16] Hastings WJ, Shalev I, Belsky DW. Translating measures of biological aging to test effectiveness of geroprotective interventions: what can we learn from research on telomeres? [Perspective]. Frontiers in Genetics. 2017;8. 10.3389/fgene.2017.00164. 10.3389/fgene.2017.00164 PMC5702647 29213278

[17] Blasco MA. Telomeres and human disease: ageing, cancer and beyond. Nat Rev Genet. 2005;6(8):611–22. 10.1038/nrg1656. 16136653 10.1038/nrg1656

[18] Martínez P, Blasco MA. Replicating through telomeres: a means to an end. Trends Biochem Sci. 2015;40(9):504–15. 10.1016/j.tibs.2015.06.003. 26188776 10.1016/j.tibs.2015.06.003

[19] Martínez P, Blasco MA. Heart-Breaking Telomeres. Circ Res. 2018;123(7):787–802. 10.1161/circresaha.118.312202. 30355079 10.1161/CIRCRESAHA.118.312202

[20] Olovnikov AM. A theory of marginotomy: The incomplete copying of template margin in enzymic synthesis of polynucleotides and biological significance of the phenomenon. J Theor Biol. 1973;41(1):181–90. 10.1016/0022-5193(73)90198-7. 4754905 10.1016/0022-5193(73)90198-7

[21] Bonnell E, Pasquier E, Wellinger RJ. Telomere Replication: Solving Multiple End Replication Problems. Front Cell Dev Biol. 2021;9:668171. 10.3389/fcell.2021.668171. 33869233 10.3389/fcell.2021.668171 PMC8047117

[22] Victorelli S, Passos JF. Telomeres and Cell Senescence. - Size Matters Not EBioMedicine. 2017;21:14–20. 10.1016/j.ebiom.2017.03.027. 28347656 10.1016/j.ebiom.2017.03.027 PMC5514392

[23] Hayflick L. THE LIMITED IN VITRO LIFETIME OF HUMAN DIPLOID CELL STRAINS. Exp Cell Res. 1965;37:614–36. 10.1016/0014-4827(65)90211-9. 14315085 10.1016/0014-4827(65)90211-9

[24] Eppard M, Passos JF, Victorelli S. Telomeres, cellular senescence, and aging: past and future. Biogerontology. 2024;25(2):329–39. 10.1007/s10522-023-10085-4. 38150087 10.1007/s10522-023-10085-4 PMC11287966

[25] Turner KJ, Vasu V, Griffin DK. Telomere biology and human phenotype. Cells. 2019;8(1). https://mdpi-res.com/d_attachment/cells/cells-08-00073/article_deploy/cells-08-00073.pdf?version=1547886840. 10.3390/cells8010073 PMC6356320 30669451

[26] Brouilette SW, Moore JS, McMahon AD, Thompson JR, Ford I, Shepherd J, Packard CJ, Samani NJ. Telomere length, risk of coronary heart disease, and statin treatment in the West of Scotland Primary Prevention Study: a nested case-control study. Lancet. 2007;369(9556):107–14. 10.1016/s0140-6736(07)60071-3. 17223473 10.1016/S0140-6736(07)60071-3

[27] Khan S, Chuturgoon AA, Naidoo DP. Telomeres and atherosclerosis. Cardiovasc J Afr. 2012;23(10):563–71. 10.5830/cvja-2012-056. 23192261 10.5830/CVJA-2012-056 PMC3721896

[28] Lung FW, Ku CS, Kao WT. Telomere length may be associated with hypertension. J Hum Hypertens. 2008;22(3):230–2. 10.1038/sj.jhh.1002314. 18046431 10.1038/sj.jhh.1002314

[29] Ma H, Zhou Z, Wei S, Liu Z, Pooley KA, Dunning AM, Svenson U, Roos G, Hosgood HD III, Shen M, Wei Q. Shortened Telomere Length Is Associated with Increased Risk of Cancer: A Meta-Analysis. PLoS ONE. 2011;6(6):e20466. 10.1371/journal.pone.0020466. 21695195 10.1371/journal.pone.0020466 PMC3112149

[30] Zhao J, Zhu Y, Lin J, Matsuguchi T, Blackburn E, Zhang Y, Cole SA, Best LG, Lee ET, Howard BV. Short leukocyte telomere length predicts risk of diabetes in american indians: the strong heart family study. Diabetes. 2014;63(1):354–62. 10.2337/db13-0744. 23949319 10.2337/db13-0744 PMC3868043

[31] Tunnicliffe L, Muzambi R, Bartlett JW, Howe LD, Basit KA, Asare K, Gore-Langton G, Mansfield KE, Codd V, Warren-Gash C. Infection and telomere length: A systematic review. PLoS ONE. 2025;20(9):e0333107. 10.1371/journal.pone.0333107. 40986533 10.1371/journal.pone.0333107 PMC12456831

[32] Page MJ, McKenzie JE, Bossuyt PM, Boutron I, Hoffmann TC, Mulrow CD, Shamseer L, Tetzlaff JM, Akl EA, Brennan SE, Chou R, Glanville J, Grimshaw JM, Hróbjartsson A, Lalu MM, Li T, Loder EW, Mayo-Wilson E, McDonald S, Moher D. The PRISMA 2020 statement: an updated guideline for reporting systematic reviews. BMJ. 2021;372:n71. 10.1136/bmj.n71. 10.1136/bmj.n71 PMC8005924 33782057

[33] Guyatt G, Oxman AD, Akl EA, Kunz R, Vist G, Brozek J, Norris S, Falck-Ytter Y, Glasziou P, deBeer H, Jaeschke R, Rind D, Meerpohl J, Dahm P, Schünemann HJ. GRADE guidelines: 1. Introduction—GRADE evidence profiles and summary of findings tables. J Clin Epidemiol. 2011;64(4):383–94. 10.1016/j.jclinepi.2010.04.026. 21195583 10.1016/j.jclinepi.2010.04.026

[34] Li L, Yu F, Yang S, Li H, Tang Y, Ma C. Lower immune senescence of T cell subsets among virologically suppressed Chinese men who have sex with men living with HIV in comparison with those ART naive. BMC Infect Dis. 2025;25(1):290. 10.1186/s12879-025-10511-7. 40021989 10.1186/s12879-025-10511-7 PMC11869689

[35] Hsieh AYY, Cai R, Bernard NF, Tremblay CL, Côté HCF. Evidence of greater immune aging among untreated HIV slow progressors than antiretroviral-controlled people living with HIV. J Infect. 2025;91(1):106511. 10.1016/j.jinf.2025.106511. 40398500 10.1016/j.jinf.2025.106511

[36] Yang NY, Hsieh AYY, Chen Z, Campbell AR, Gadawski I, Kakkar F, Sauvé L, Bitnun A, Brophy J, Murray MCM. Chronic and latent viral infections and leukocyte telomere length across the lifespan of female and male individuals living with or without HIV [Article]. Viruses. 2024;16(5):755. 10.3390/v16050755. 10.3390/v16050755 PMC11125719 38793637

[37] Shiau S, Zumpano F, Wang Z, Shah J, Tien PC, Ross RD, Sharma A, Yin MT. Epigenetic Aging and Musculoskeletal Outcomes in a Cohort of Women Living With HIV. J Infect Dis. 2024;229(6):1803–11. 10.1093/infdis/jiae016. 38366369 10.1093/infdis/jiae016 PMC11175700

[38] Sehl ME, Breen EC, Shih R, Li F, Zhang J, Langfelder P, Horvath S, Bream JH, Duggal P, Martinson J. Decreased but persistent epigenetic age acceleration is associated with changes in T-cell subsets after initiation of highly active antiretroviral therapy in persons living with HIV [Article]. Front Bioinform. 2024;4:1356509. 10.3389/fbinf.2024.1356509. 10.3389/fbinf.2024.1356509 PMC11157435 38855141

[39] Petrara MR, Ruffoni E, Carmona F, Cavallari I, Zampieri S, Morello M, Del Bianco P, Rampon O, Cotugno N, Palma P, Rossi P, Giaquinto C, Giunco S, De Rossi A. HIV reservoir and premature aging: risk factors for aging-associated illnesses in adolescents and young adults with perinatally acquired HIV. PLoS Pathog. 2024;20(9):e1012547. 10.1371/journal.ppat.1012547. 39312589 10.1371/journal.ppat.1012547 PMC11449303

[40] Macamo ED, Mkhize-Kwitshana ZL, Duma Z, Mthombeni J, Naidoo P. Telomere Length in a South African Population Co-Infected with HIV and Helminths. Curr Issues Mol Biol. 2024;46(7):6853–67. 10.3390/cimb46070409. 39057051 10.3390/cimb46070409 PMC11276263

[41] Liang X, Aouizerat BE, So-Armah K, Cohen MH, Marconi VC, Xu K, Justice AC. DNA methylation-based telomere length is associated with HIV infection, physical frailty, cancer, and all-cause mortality. Aging Cell. 2024;23(7):e14174. 10.1111/acel.14174. 38629454 10.1111/acel.14174 PMC11258465

[42] Cadinanos J, Rodríguez-Centeno J, Montejano R, Esteban-Cantos A, Mena-Garay B, Jiménez-González M, Saiz-Medrano G, de Miguel R, Artalejo R, F., Bernardino JI. Partial recovery of telomere length after long-term virologic suppression in persons with HIV-1 [Article]. Open Forum Infect Dis. 2024;11(10):ofae550. 10.1093/ofid/ofae550. 10.1093/ofid/ofae550 PMC11482007 39416992

[43] Bukic E, Lukić G, Toljić B, Obradovic B, Jadžić J, Jevtović D, Milasin JM. TERT single nucleotide polymorphism rs2736098 but not rs2736100 is associated with telomere length in HIV-infected patients on cART [Article]. Mol Biol Rep. 2024;51(1):147. 10.1007/s11033-023-08967-4. 10.1007/s11033-023-08967-4 38236501

[44] Van Ommen CE, Hsieh AYY, Albert AY, Kimmel ER, Cote HCF, Maan EJ, Prior JC, Pick N, Murray MCM. Lower anti-Müllerian hormone levels are associated with HIV in reproductive age women and shorter leukocyte telomere length among late reproductive age women. Aids. 2023;37(5):769–78. 10.1097/qad.0000000000003481. 36726239 10.1097/QAD.0000000000003481 PMC9994852

[45] Toljić B, Milasin J, De Luka SR, Lukić G, Jevtović D, Maslać A, Ristić-Djurović JL, Trbovich AM. HIV-infected patients as a model of aging [Article]. Microbiol Spectr. 2023;11(3). 10.1128/spectrum.00532-23. 10.1128/spectrum.00532-23 PMC10269491 37093018

[46] Lombardi F, Sanfilippo A, Fabbiani M, Borghetti A, Ciccullo A, Tamburrini E, Di Giambenedetto S. Blood telomere length gain in people living with HIV switching to dolutegravir plus lamivudine versus continuing triple regimen: a longitudinal, prospective, matched, controlled study. J Antimicrob Chemother. 2023;78(9):2315–22. 10.1093/jac/dkad237. 37534393 10.1093/jac/dkad237 PMC10477130

[47] Bukic E, Milasin J, Toljić B, Jadžić J, Jevtović D, Obradovic B, Lukić G. Association between combination antiretroviral therapy and telomere length in people living with human immunodeficiency virus [Article]. Biology. 2023;12(9):1210. 10.3390/biology12091210. 10.3390/biology12091210 PMC10525818 37759609

[48] Sehl ME, Breen EC, Shih R, Chen L, Wang R, Horvath S, Bream JH, Duggal P, Martinson J, Wolinsky SM. Increased rate of epigenetic aging in men living with HIV prior to treatment [Article]. Front Genet. 2022;12:796547. 10.3389/fgene.2021.796547. 10.3389/fgene.2021.796547 PMC8919029 35295196

[49] Schoepf IC, Thorball CW, Ledergerber B, Kootstra NA, Reiss P, Raffenberg M, Engel T, Braun DL, Hasse B, Thurnheer C. Telomere Length Declines in Persons With Human Immunodeficiency Virus Before Antiretroviral Therapy Start but Not After Viral Suppression: A Longitudinal Study Over > 17 Years [Article]. J Infect Dis. 2022;225(9):1581–91. 10.1093/infdis/jiab603. 34910812 10.1093/infdis/jiab603

[50] Rodríguez-Centeno J, Esteban-Cantos A, Montejano R, Stella-Ascariz N, de Miguel R, Mena-Garay B, Saiz-Medrano G, Alejos B, Jiménez-González M, Bernardino JI. Effects of tenofovir on telomeres, telomerase and T cell maturational subset distribution in long-term aviraemic HIV-infected adults [Article]. J Antimicrob Chemother. 2022;77(4):1125–32. 10.1093/jac/dkab492. 35045162 10.1093/jac/dkab492

[51] Breen EC, Sehl ME, Shih R, Langfelder P, Wang R, Horvath S, Bream JH, Duggal P, Martinson J, Wolinsky SM, Martínez-Maza O, Ramirez CM, Jamieson BD. Accelerated aging with HIV begins at the time of initial HIV infection. iScience. 2022;25(7):104488. 10.1016/j.isci.2022.104488. 35880029 10.1016/j.isci.2022.104488 PMC9308149

[52] Shiau S, Arpadi SM, Shen Y, Cantos A, Ramon CV, Shah J, Jang G, Manly JJ, Brickman AM, Baccarelli AA. Epigenetic Aging Biomarkers Associated With Cognitive Impairment in Older African American Adults With Human Immunodeficiency Virus (HIV) [Article]. Clin Infect Dis. 2021;73(11):1982–91. 10.1093/cid/ciab563. 34143869 10.1093/cid/ciab563 PMC8664485

[53] Raffenberg M, Engel T, Schoepf IC, Kootstra NA, Reiss P, Braun DL, Thorball CW, Fellay J, Kouyos RD, Ledergerber B, Günthard HF, Tarr PE. Impact of Delaying Antiretroviral Treatment During Primary Human Immunodeficiency Virus Infection on Telomere Length. J Infect Dis. 2021;224(10):1775–84. 10.1093/infdis/jiab186. 33822976 10.1093/infdis/jiab186

[54] Mehta SR, Iudicello JE, Lin J, Ellis RJ, Morgan E, Okwuegbuna O, Cookson D, Karris M, Saloner R, Heaton R, Grant I, Letendre S. Telomere length is associated with HIV infection, methamphetamine use, inflammation, and comorbid disease risk. Drug Alcohol Depend. 2021;221:108639. 10.1016/j.drugalcdep.2021.108639. 33621803 10.1016/j.drugalcdep.2021.108639 PMC8026664

[55] Engel T, Raffenberg M, Schoepf IC, Kootstra NA, Reiss P, Thorball CW, Hasse B, Hirzel C, Wissel K, Roth JA. Telomere Length, Traditional Risk Factors, Factors Related to Human Immunodeficiency Virus (HIV) and Coronary Artery Disease Events in Swiss Persons Living With HIV [Article]. Clin Infect Dis. 2021;73(7):E2070–6. 10.1093/cid/ciaa1034. 32725240 10.1093/cid/ciaa1034

[56] Iyengar S, Cȏté HCF, Fitch KV, Torriani M, Feldpausch M, Srinivasa S. Relationship of telomere length to fat redistribution in HIV. Open Forum Infect Dis. 2020;7(12). 10.1093/ofid/ofaa523. 10.1093/ofid/ofaa523 PMC7733235 33335933

[57] Hsieh AYY, Kimmel E, Pick N, Sauvé L, Brophy J, Kakkar F, Bitnun A, Murray MCM, Côté HC F. Inverse relationship between leukocyte telomere length attrition and blood mitochondrial DNA content loss over time [Article]. Aging. 2020;12:1–26. 10.18632/AGING.103703. 32703912 10.18632/aging.103703 PMC7467389

[58] Chalouni M, Rodríguez-Centeno J, Samri A, Blanco J, Stella-Ascariz N, Wallet C, Knobel H, Zucman D, Ferreras BA, Autran B. Correlation between blood telomere length and CD4 + CD8+ T-cell subsets changes 96 weeks after initiation of antiretroviral therapy in HIV-1-positive individuals [Article]. PLoS ONE. 2020;15(4):e0230772. 10.1371/journal.pone.0230772. 10.1371/journal.pone.0230772 PMC7141657 32267847

[59] Wang S, Chang E, Byanyima P, Huang P, Sanyu I, Musisi E, Sessolo A, Davis JL, Worodria W, Huang L. Association between common telomere length genetic variants and telomere length in an African population and impacts of HIV and TB [Article]. J Hum Genet. 2019;64(10):1033–40. 10.1038/s10038-019-0646-9. 31388112 10.1038/s10038-019-0646-9 PMC7039711

[60] Saberi S, Kalloger SE, Zhu MMT, Sattha B, Maan EJ, van Schalkwyk J, Money DM, Côté HCF. Dynamics of leukocyte telomere length in pregnant women living with HIV, and HIV-negative pregnant women: A longitudinal observational study [Article]. PLoS ONE. 2019;14(3):e0212273. 10.1371/journal.pone.0212273. 30840638 10.1371/journal.pone.0212273 PMC6402636

[61] Paghera S, Quiròs-Roldan E, Sottini A, Properzi M, Castelli F, Imberti L. Lymphocyte homeostasis is maintained in perinatally HIV-infected patients after three decades of life [Article]. Immun Ageing. 2019;16(1):26. 10.1186/s12979-019-0166-7. 10.1186/s12979-019-0166-7 PMC6791008 31636688

[62] Minami R, Takahama S, Yamamoto M. Correlates of telomere length shortening in peripheral leukocytes of HIV-infected individuals and association with leukoaraiosis [Article]. PLoS ONE. 2019;14(6):e0218996. 10.1371/journal.pone.0218996. 31246986 10.1371/journal.pone.0218996 PMC6597162

[63] Babu H, Ambikan AT, Gabriel EE, Svensson-Akusjärvi SS, Palaniappan AN, Sundaraj V, Mupanni NR, Sperk M, Cheedarla N, Sridhar R, Tripathy SP, Nowak P, Hanna LE, Neogi U. Systemic inflammation and the increased risk of inflamm-aging and age-associated diseases in people living with HIV on long term suppressive antiretroviral therapy [Article]. Front Immunol. 2019;10(AUG):1965. 10.3389/fimmu.2019.01965. 10.3389/fimmu.2019.01965 PMC6718454 31507593

[64] Alejos B, Stella-Ascariz N, Montejano R, Rodríguez-Centeno J, Schwimmer C, Bernardino JI, Rodès B, Esser S, Goujard C, Sarmento-Castro R. Determinants of blood telomere length in antiretroviral treatment-naïve HIV-positive participants enrolled in the NEAT 001/ANRS 143 clinical trial [Article]. HIV Med. 2019;20(10):691–8. 10.1111/hiv.12791. 31532902 10.1111/hiv.12791

[65] Stella-Ascariz N, Montejano R, Rodriguez-Centeno J, Alejos B, Schwimmer C, Bernardino JI, Rodes B, Allavena C, Hoffmann C, Gisslén M, de Miguel R, Esteban-Cantos A, Wallet C, Raffi F, Arribas JR, Group NAS. Blood Telomere Length Changes After Ritonavir-Boosted Darunavir Combined With Raltegravir or Tenofovir-Emtricitabine in Antiretroviral-Naive Adults Infected With HIV-1. J Infect Dis. 2018;218(10):1523–30. 10.1093/infdis/jiy399. 29982509 10.1093/infdis/jiy399

[66] Montejano R, Stella-Ascariz N, Monge S, Bernardino JI, Pérez-Valero I, Montes ML, Valencia E, Martín-Carbonero L, Moreno V, González-García J, Rodríguez-Centeno J, Rodès B, Cantos AE, Alejos B, de Miguel R, Arnalich-Fernández F, Perona R, Arribas JR. Impact of Nucleos(t)ide Reverse Transcriptase Inhibitors on Blood Telomere Length Changes in a Prospective Cohort of Aviremic HIV-Infected Adults [Article]. J Infect Dis. 2018;218(10):1531–40. 10.1093/infdis/jiy364. 29912427 10.1093/infdis/jiy364

[67] Kalyan S, Pick N, Mai A, Murray MCM, Kidson K, Chu J, Albert AY, Côté HCF, Maan EJ, Goshtasebi A, Money DM, Prior JC. Premature spinal bone loss in women living with hiv is associated with shorter leukocyte telomere length [Article]. International Journal of Environmental Research and Public Health. 2018;15(5):1018. 10.3390/ijerph15051018. 10.3390/ijerph15051018 PMC5982057 29783641

[68] Ding Y, Lin H, Zhou S, Wang K, Li L, Zhang Y, Yao Y, Gao M, Liu X, He N. Stronger association between insomnia symptoms and shorter telomere length in old HIV-infected patients compared with uninfected individuals [Article]. Aging Disease. 2018;9(6):1010–9. 10.14336/AD.2018.0204. 30574414 10.14336/AD.2018.0204 PMC6284770

[69] de Araújo ML, Duarte W, de Oliveira ACP, Gascon MRP, Fonseca LAM, Paiva RMA, Santana B, Calado RT, Casseb J. Is the telomere length associated with neurocognitive disabilities in HIV-1-infected subjects? [Article]. Rev Inst Med Trop Sao Paulo. 2018;60:e16. 10.1590/S1678-9946201860016. 10.1590/S1678-9946201860016 PMC5956918 29694602

[70] Montejano R, Stella-Ascariz N, Monge S, Bernardino JI, Pérez-Valero I, Montes ML, Valencia E, Martín-Carbonero L, Moreno V, González-García J. (2017). Impact of antiretroviral treatment containing tenofovir difumarate on the telomere length of aviremic HIV-infected patients [Article]. J Acquir Immune Defic Syndr. 1999;76(1):102–109. 10.1097/QAI.0000000000001391. 10.1097/QAI.0000000000001391 28418989

[71] Leung JM, Fishbane N, Jones M, Morin A, Xu S, Liu JC, MacIsaac J, Milloy MJ, Hayashi K, Montaner J, Horvath S, Kobor M, Sin DD, Harrigan PR, Man SF. Longitudinal study of surrogate aging measures during human immunodeficiency virus seroconversion. Aging. 2017;9(3):687–705. 10.18632/aging.101184. 28237978 10.18632/aging.101184 PMC5391226

[72] Gonzalez-Serna A, Ajaykumar A, Gadawski I, Muñoz-Fernández MA, Hayashi K, Harrigan PR, Côté HCF. Rapid Decrease in Peripheral Blood Mononucleated Cell Telomere Length After HIV Seroconversion, but Not HCV Seroconversion. J Acquir Immune Defic Syndr. 2017;76(1):e29–32. 10.1097/qai.0000000000001446. 28797026 10.1097/QAI.0000000000001446 PMC6155455

[73] Jimnez VC, Joerink M, Maurer I, Harskamp AM, van Leeuwen EMM, Booiman T, Deeks SG, Kootstra NA, Reiss P, Wit FW N. M. T-Cell Activation Independently Associates with Immune Senescence in HIV-Infected Recipients of Long-term Antiretroviral Treatment [Article]. J Infect Dis. 2016;214(2):216–25. 10.1093/infdis/jiw146. 27073222 10.1093/infdis/jiw146 PMC8445638

[74] Auld E, Lin J, Chang E, Byanyima P, Ayakaka I, Musisi E, Worodria W, Davis JL, Segal M, Blackburn E, Huang L. HIV Infection Is Associated with Shortened Telomere Length in Ugandans with Suspected Tuberculosis. PLoS ONE. 2016;11(9):e0163153. 10.1371/journal.pone.0163153. 27655116 10.1371/journal.pone.0163153 PMC5031464

[75] Liu JC, Leung JM, Ngan DA, Nashta NF, Guillemi S, Harris M, Lima VD, Um SJ, Li Y, Tam S, Shaipanich T, Raju R, Hague C, Leipsic JA, Bourbeau J, Tan WC, Harrigan PR, Sin DD, Montaner J, Man SF. Absolute leukocyte telomere length in HIV-infected and uninfected individuals: evidence of accelerated cell senescence in HIV-associated chronic obstructive pulmonary disease. PLoS ONE. 2015;10(4):e0124426. 10.1371/journal.pone.0124426. 25885433 10.1371/journal.pone.0124426 PMC4401786

[76] Blanco JR, Jarrin I, Martínez A, Siles E, Larráyoz IM, Cañuelo A, Gutierrez F, González-García J, Vidal Marsal F, Moreno S. Shorter telomere length predicts poorer immunological recovery in virologically suppressed hiv-1-infected patients treated with combined antiretroviral therapy. J Acquir Immune Defic Syndr. 2015;68(1):21–9. 10.1097/QAI.0000000000000398. [Article]. 25321176 10.1097/QAI.0000000000000398

[77] Zanet DAL, Thorne A, Singer J, Maan EJ, Sattha B, Le Campion A, Soudeyns H, Pick N, Murray M, Money DM. Association between short leukocyte telomere length and HIV infection in a cohort study: No evidence of a relationship with antiretroviral therapy [Article]. Clin Infect Dis. 2014;58(9):1322–32. 10.1093/cid/ciu051. 24457340 10.1093/cid/ciu051

[78] Srinivasa S, Fitch KV, Petrow E, Burdo TH, Williams KC, Lo J, Cȏté HCF, Grinspoon SK. Soluble CD163 is associated with shortened telomere length in HIV-infected patients. JAIDS J Acquir Immune Defic Syndr. 2014;67(4):414–8. 10.1097/qai.0000000000000329. 25197827 10.1097/QAI.0000000000000329 PMC4213298

[79] Solomon A, Tennakoon S, Leeansyah E, Arribas J, Hill A, Van Delft Y, Moecklinghoff C, Lewin SR. No difference in the rate of change in telomere length or telomerase activity in HIV-infected patients after three years of darunavir/ritonavir with and without nucleoside analogues in the MONET trial. PLoS ONE. 2014;9(11):e109718. 10.1371/journal.pone.0109718. 25368992 10.1371/journal.pone.0109718 PMC4219673

[80] Pathai S, Lawn SD, Gilbert CE, McGuinness D, McGlynn L, Weiss HA, Port J, Christ T, Barclay K, Wood R, Bekker LG, Shiels PG. Accelerated biological ageing in HIV-infected individuals in South Africa: a case-control study. Aids. 2013;27(15):2375–84. 10.1097/QAD.0b013e328363bf7f. 23751258 10.1097/QAD.0b013e328363bf7f PMC3805356

[81] Malan-Müller S, Hemmings SMJ, Spies G, Kidd M, Fennema-Notestine C, Seedat S. Shorter telomere length - a potential susceptibility factor for HIV-associated neurocognitive impairments in South African woman [Article]. PLoS ONE. 2013;8(3):e58351. 10.1371/journal.pone.0058351. 10.1371/journal.pone.0058351 PMC3589394 23472184

[82] Leeansyah E, Cameron PU, Solomon A, Tennakoon S, Velayudham P, Gouillou M, Spelman T, Hearps A, Fairley C, Smit de V, Pierce AB, Armishaw J, Crowe SM, Cooper DA, Koelsch KK, Liu JP, Chuah J, Lewin SR. Inhibition of telomerase activity by human immunodeficiency virus (HIV) nucleos(t)ide reverse transcriptase inhibitors: a potential factor contributing to HIV-associated accelerated aging. J Infect Dis. 2013;207(7):1157–65. 10.1093/infdis/jit006. 23303810 10.1093/infdis/jit006

[83] Rickabaugh TM, Kilpatrick RD, Hultin LE, Hultin PM, Hausner MA, Sugar CA, Althoff KN, Margolick JB, Rinaldo CR, Detels R. The dual impact of HIV-1 infection and aging on naïve CD4 + T-cells: Additive and distinct patterns of impairment [Article]. PLoS ONE. 2011;6(1):e16459. 10.1371/journal.pone.0016459. 10.1371/journal.pone.0016459 PMC3027697 21298072

[84] Tucker V, Jenkins J, Gilmour J, Savoie H, Easterbrook P, Gotch F, Browning MJ. T-cell telomere length maintained in HIV-infected long-term survivors. HIV Med. 2000;1(2):116–22. 10.1046/j.1468-1293.2000.00010.x. 11737334 10.1046/j.1468-1293.2000.00010.x

[85] Richardson MW, Sverstiuk A, Hendel H, Cheung TW, Zagury JF, Rappaport J. Analysis of telomere length and thymic output in fast and slow/non-progressors with HIV infection. Biomed Pharmacother. 2000;54(1):21–31. 10.1016/s0753-3322(00)88637-0. 10721459 10.1016/s0753-3322(00)88637-0

[86] Bestilny LJ, Gill MJ, Mody CH, Riabowol KT. Accelerated replicative senescence of the peripheral immune system induced by HIV infection [Article]. Aids. 2000;14(7):771–80. 10.1097/00002030-200005050-00002. 10839584 10.1097/00002030-200005050-00002

[87] Wolthers KC, Noest AJ, Otto SA, Miedema F, De Boer RJ. Normal telomere lengths in naive and memory CD4 + T cells in HIV type 1 infection: A mathematical interpretation [Article]. AIDS Res Hum Retroviruses. 1999;15(12):1053–62. 10.1089/088922299310340. 10461825 10.1089/088922299310340

[88] Nichols WS, Schneider S, Chan RCK, Farthing CF, Daar ES. Increased CD4 + T-lymphocyte senescence fraction in advanced human immunodeficiency virus type 1 infection [Article]. Scand J Immunol. 1999;49(3):302–6. 10.1046/j.1365-3083.1999.00505.x. 10102648 10.1046/j.1365-3083.1999.00505.x

[89] Kaushal S, Landay AL, Lederman MM, Connick E, Spritzler J, Kuritzkes DR, Kessler H, Levine BL, St. Louis DC, June CH. Increases in T Cell Telomere Length in HIV Infection after Antiretroviral Combination Therapy for HIV-1 Infection Implicate Distinct Population Dynamics in CD4 + and CD8 + T Cells. Clin Immunol. 1999;92(1):14–24. 10.1006/clim.1999.4726. 10413649 10.1006/clim.1999.4726

[90] Feng YR, Biggar RJ, Gee D, Norwood D, Zeichner SL, Dimitrov DS. Long-term telomere dynamics: Modest increase of cell turnover in HIV-infected individuals followed for up to 14 years [Article]. Pathobiology. 1999;67(1):34–8. 10.1159/000028048. 9873226 10.1159/000028048

[91] Palmer LD, Weng NP, Levine BL, June CH, Lane HC, Hodes RJ. Telomere length, telomerase activity, and replicative potential in HIV infection: Analysis of CD4 + and CD8 + T cells from HIV-discordant monozygotic twins [Article]. J Exp Med. 1997;185(7):1381–6. 10.1084/jem.185.7.1381. 9104824 10.1084/jem.185.7.1381 PMC2196247

[92] Effros RB, Allsopp R, Chiu CP, Hausner MA, Hirji KI, Wang L, Harley CB, Villeponteau B, West MD, Giorgi JV. Shortened telomeres in the expanded CD28- CD8 + cell subset in HIV disease implicate replicative senescence in HIV pathogenesis [Article]. Aids. 1996;10(8):F17–22. 10.1097/00002030-199607000-00001. 8828735 10.1097/00002030-199607000-00001

[93] Huzen J, Wong LSM, van Veldhuisen DJ, Samani NJ, Zwinderman AH, Codd V, Cawthon RM, Benus GFJD, van der Horst ICC, Navis G, Bakker SJL, Gansevoort RT, de Jong PE, Hillege HL, van Gilst WH, de Boer RA, van der Harst P. Telomere length loss due to smoking and metabolic traits. J Intern Med. 2014;275(2):155–63. 10.1111/joim.12149. 24118582 10.1111/joim.12149

[94] Lim Y-S, Nguyen MTN, Pham TX, Huynh TTX, Park E-M, Choi DH, Kang SM, Tark D, Hwang SB. Hepatitis C Virus Nonstructural 5A Protein Interacts with Telomere Length Regulation Protein: Implications for Telomere Shortening in Patients Infected with HCV. Mol Cells. 2022;45(3):148–57. 10.14348/molcells.2021.0167. 34949741 10.14348/molcells.2021.0167 PMC8926864

[95] Lv T, Cao W, Li T. HIV-Related Immune Activation and Inflammation: Current Understanding and Strategies. J Immunol Res. 2021;2021(1):7316456. 10.1155/2021/7316456. 34631899 10.1155/2021/7316456 PMC8494587

[96] Gardner M, Bann D, Wiley L, Cooper R, Hardy R, Nitsch D, Martin-Ruiz C, Shiels P, Sayer AA, Barbieri M, Bekaert S, Bischoff C, Brooks-Wilson A, Chen W, Cooper C, Christensen K, De Meyer T, Deary I, Der G, Ben-Shlomo Y. Gender and telomere length: Systematic review and meta-analysis. Exp Gerontol. 2014;51:15–27. 10.1016/j.exger.2013.12.004. 24365661 10.1016/j.exger.2013.12.004 PMC4523138

[97] Mayer S, Brüderlein S, Perner S, Waibel I, Holdenried A, Ciloglu N, Hasel C, Mattfeldt T, Nielsen KV, Möller P. Sex-specific telomere length profiles and age-dependent erosion dynamics of individual chromosome arms in humans. Cytogenet Genome Res. 2006;112(3–4):194–201. 10.1159/000089870. 16484772 10.1159/000089870

[98] Panelli DM, Bianco K. Cellular aging and telomere dynamics in pregnancy. Curr Opin Obstet Gynecol. 2022;34(2):57–61. 10.1097/gco.0000000000000765. 34845136 10.1097/GCO.0000000000000765 PMC8891073

[99] Sluis-Cremer N, Tachedjian G. Mechanisms of inhibition of HIV replication by non-nucleoside reverse transcriptase inhibitors. Virus Res. 2008;134(1–2):147–56. 10.1016/j.virusres.2008.01.002. 18372072 10.1016/j.virusres.2008.01.002 PMC2745993

[100] Rubtsova MP, Vasilkova DP, Malyavko AN, Naraikina YV, Zvereva MI, Dontsova OA. Telomere lengthening and other functions of telomerase. Acta Naturae. 2012;4(2):44–61. 22872811 PMC3408703

[101] Peng Y, Mian IS, Lue NF. Analysis of Telomerase Processivity: Mechanistic Similarity to HIV-1 Reverse Transcriptase and Role in Telomere Maintenance. Mol Cell. 2001;7(6):1201–11. 10.1016/S1097-2765(01)00268-4. 11430823 10.1016/s1097-2765(01)00268-4

[102] Fastenackels S, Sauce D, Vigouroux C, Avettand-Fènoël V, Bastard JP, Fellahi S, Nailler L, Arezes E, Rouzioux C, Warszawski J, Viard JP, Appay V. HIV-mediated immune aging in young adults infected perinatally or during childhood. Aids. 2019;33(11):1705–10. 10.1097/qad.0000000000002275. 31149945 10.1097/QAD.0000000000002275

[103] Frank AP, de Souza Santos R, Palmer BF, Clegg DJ. Determinants of body fat distribution in humans may provide insight about obesity-related health risks. J Lipid Res. 2019;60(10):1710–9. 10.1194/jlr.R086975. 30097511 10.1194/jlr.R086975 PMC6795075

