# Supplementary material for: Telomere length dynamics in adults living with HIV: A systematic review
Source: BMC Infect Dis. 2026 Apr 18;26:1062. doi: 10.1186/s12879-026-13243-4 (PMC13227684; doi:10.1186/s12879-026-13243-4)
Supplement: Supplementary file 1 — Supplementary Material 1 [file 12879_2026_13243_MOESM1_ESM.docx]

# **Supplementary Material**

**Supplementary Table 1:** Searches conducted on 07/03/2026

| **Database** | **Date of search** | **Search fields** | **Search string** | **Filters applied** | **Results** |
| --- | --- | --- | --- | --- | --- |
| Pubmed | 25/09/2025 | Title/Abstract | ("telomere length"[Title/Abstract] OR "telomere shortening"[Title/Abstract] OR "telomere attrition"[Title/Abstract] OR "telomeres"[Title/Abstract]) AND ("HIV"[Title/Abstract] OR "human immunodeficiency virus"[Title/Abstract] OR "PLWH"[Title/Abstract]) | **Date:**1996-2025  **Species:** Human studies  **Language:** English | 186 |
| Scopus | 25/09/2025 | Title/Abstract/  Keywords | "telomere length" OR "telomere shortening" OR "telomere attrition" OR “telomeres” AND “HIV” OR "human immunodeficiency virus" OR “PLWH” | **Date:** 1996-2025  **Document type:** Articles  **Language:** English | 1688 |
| Science Direct | 25/09/2025 | Search Terms | ("telomere length" OR "telomere shortening" OR telomeres OR "telomere attrition") AND (HIV OR "human immunodeficiency virus" OR PLWH) | **Date:** 1996-2025  **Article type:** Research Articles  **Languages:** English | 1220 |
| Google Scholar | 25/09/2025 | Title | allintitle: "telomere length" and "HIV" | **Date:** 1996-2025 | 64 |
